# Precise delivery of doxorubicin and imiquimod through pH-responsive tumor microenvironment-active targeting micelles for chemo- and immunotherapy

**DOI:** 10.1016/j.mtbio.2022.100482

**Published:** 2022-11-03

**Authors:** Yu-Han Wen, Po-I Hsieh, Hsin-Cheng Chiu, Chil-Wei Chiang, Chun-Liang Lo, Yi-Ting Chiang

**Affiliations:** a Department of Biomedical Engineering, National Yang Ming Chiao Tung University, Taipei, 112, Taiwan; b Medical Device Innovation and Translation Center, National Yang Ming Chiao Tung University, Taipei, 112, Taiwan; c Department of Biomedical Engineering and Environmental Sciences, National Tsing-Hua University, Hsinchu, 300, Taiwan; d School of Pharmacy, China Medical University, Taichung, 406040, Taiwan

**Keywords:** Chemotherapy, Immunotherapy, Tumor microenvironment, pH-sensitive, Micelles

## Abstract

Recently, combining immunotherapy and chemotherapy has become a promising strategy to treat cancer. However, this therapeutic strategy still has its limitations because of the adverse effects caused by the simultaneous administration of multiple therapeutic agents. Using nanoparticles is an effective approach to successfully combine these therapies because they can reduce side effects, increase circulation time, and ensure the delivery of cytotoxic agents to tumor tissues. In this study, dual pH-sensitive and tumor microenvironment (TME)-active targeting micelles comprising poly(propyl methacrylate-*co*-glucosamine/histidine/doxorubicin) (P(PAA-*co*-GLU/HIS/DOX) and methoxy-poly(ethylene glycol)-*block*-poly(l-lysine) were prepared to encapsulate an immunomodulator, imiquimod (IMQ). Because these micelles can expose glucose targeting ligands at the TME and pH-dependently release IMQ and DOX, micelles effectively inhibit the growth of 4T1 cells selectively and highly accumulate in 4T1 cells as the pH decreased to 6.5. Moreover, in RAW 264.7 ​cells, these micelles prevent cell death and induce M1 macrophage polarization. In 4T1 orthotopic tumor-bearing mice, micelles not only exhibited high tumor accumulation, effective tumor inhibition, and fewer adverse effects, but also dramatically increased the number of mature dendritic cells, activate cytotoxic T cells, and polarize M1-like macrophages in tumor tissues. Overall, these micelles exhibit precise pH responsiveness and ideal drug delivery capabilities for combined chemo- and immunotherapy; these results significantly contribute to the future development of nanomedicines in cancer therapy.

## Highlights

• Micelles exhibit stepwise drug-release behavior and cancer-targeting ability in the TME.
• IMQ released in the TME polarizes TAMs and activates immune cells for cancer immunotherapy.
• DOX was released in the endosomes/secondary lysosomes of cancer cells for chemotherapy.

## Graphical abstract

A multifunctional micelle is designed for exposing targeting ligands and releasing partial imiquimod at the tumor microenvironment, thereby inducing tumor accumulation, cancer cell cytotoxicity, M1-type macrophage polarization, and immune cell activation for cancer chemo- and immunotherapy.

**Figure:**
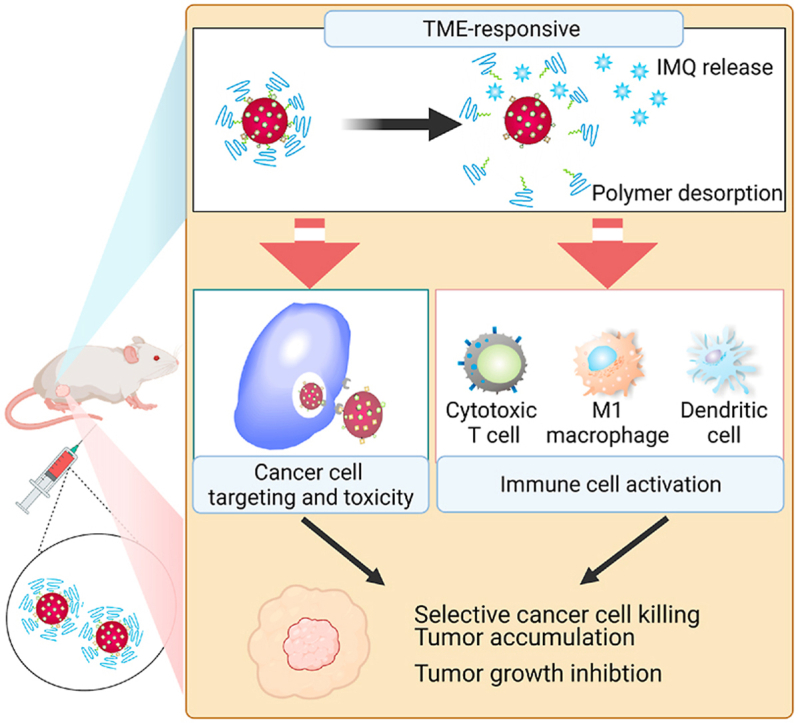

## 1 Introduction

Malignant neoplasms are deadly diseases that take millions of lives around the world. Conventional treatments including surgery, chemotherapy, and radiation are commonly used for cancer therapy. However, these therapeutic strategies often cause adverse effects, making it necessary to develop an efficient way to treat cancer. In recent years, several immunotherapeutic products have been approved for use in cancer [1]. However, developing a new drug is riskier and has a higher cost than developing a drug delivery system or a new combined therapeutic strategy. Compared to an entirely new structure drug application, through 505(b) (2) new drug applications (NDAs) allow the use of published experimental results to avoid repeating studies and offer accelerated nonclinical testing programs [2]. Targeting the tumor microenvironment (TME) is a prudent strategy to not only minimize adverse effects during the treatment, but also improve therapeutic efficacy [[3], [4], [5]]. Numerous studies focused on drug delivery to the TME have reported dramatic tumor growth inhibition by a combination of immunotherapy with chemotherapy [6], radiation [7], or photothermal therapy [8].

The TME comprises cancer cells and various immunosuppressive cells, such as myeloid-derived suppressor cells (MDSCs), tumor-associated macrophages (TAMs), and T regulatory cells (Tregs) [9]. Cancer cells have a survival feature to evade immune recognition and exclusion [10]. Tumor immunosuppressive cells also contribute to immunosuppression and immune escape [9]. Activation of dendritic cells (DCs) can be useful in cancer treatments because they are crucial in linking the innate and adaptive immune responses [11]. Imiquimod (IMQ), a Toll-like receptor 7 (TLR-7) agonist, was used to treat actinic keratosis (AK) and superficial basal cell carcinoma (BCC) [12]. Once IMQ interacts with TLR-7 in the intracellular endosomes, DCs can begin eliciting cytokines and chemokines including IFNα, TNF-α, IL-2, IL-6, IL12, G-CSF, and GM-CSF. DCs could then be activated to attract mature cytotoxic T-lymphocytes and other immune cells into the tumor tissues, especially through transcription factors for NF-kB [13,14]. Moreover, IMQ has been shown to decrease Tregs recruitment and potentially switch TAMs into M1-like macrophages in the tumor tissue [15,16], resulting in tumor growth inhibition [17]. Although IMQ is an immunostimulant for immunotherapy, its clinical applications are highly limited because of its hydrophobicity and low tumor-targeting ability [6,16].

Nanoparticles are considered a potential therapeutic approach to improving drug dissolution, reducing adverse effects, and prolonging the circulation time of active pharmaceutical ingredients, thereby enhancing bioavailability and improving the therapeutic index [[18], [19], [20]]. Furthermore, nanoparticles are designed using materials well-suited for responding to the hallmarks of malignant neoplasms; therefore, they can specifically target and treat cancer cells. Cancer cells exhibit high glucose metabolism and consumption even in aerobic conditions through aerobic glycolysis, which is known as the “Warburg effect” [21,22]. This process also produces excess lactate, inducing a more acidic TME compared to normal tissues [23,24]. Based on these features of cancer cells, glucose-conjugating nanoparticles have been shown to have increased uptake by cancer cells, thereby exhibiting higher anti-tumor efficacy [25,26]. Moreover, controlling the release of the cargo from nanoparticles into tumor tissues or cancer cells has been achieved by developing pH-sensitive structures, such as histidine, acrylic acid [27], *cis*-aconityl amide [28], benzoic imine [29], hydrazone [30], and acetal/ketal [31] to specifically release encapsulated drugs into the TME or into intracellular endosomes/secondary lysosomes through structural protonation or hydrolysis.

**Fig. 1:**
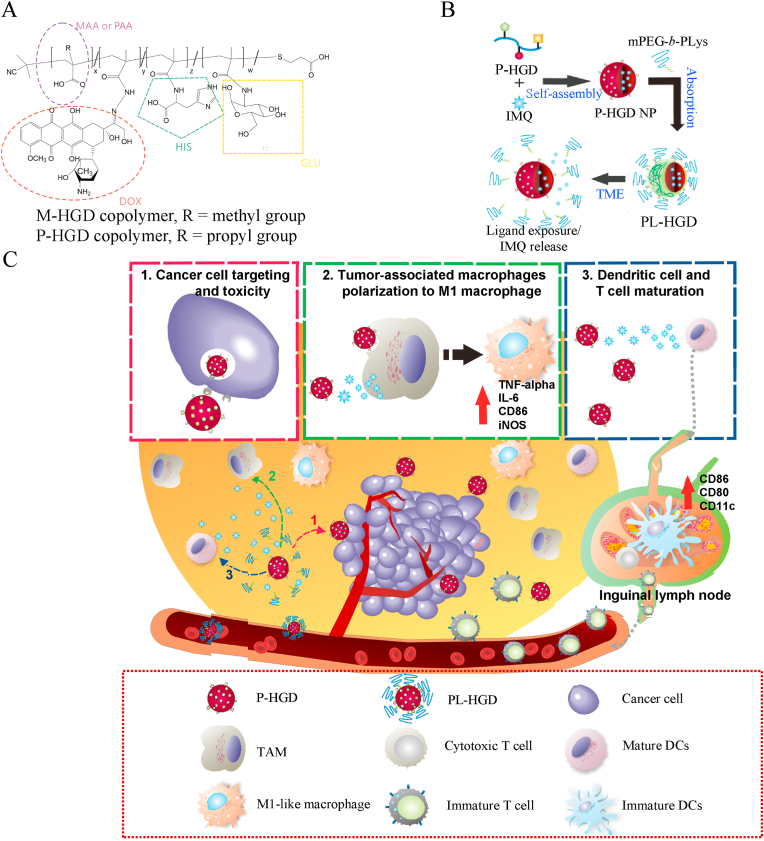
Schematic representation of the structure of copolymers and preparation process and functions of micelles. (A) The structure of M-HGD and P-HGD copolymers. (B) IMQ-containing P-HGD nanoparticles were prepared using a dialysis method. mPEG-*b*-PLys copolymers were then attached onto P-HGD nanoparticles through electrostatic interactions to form PL-HGD micelles. (C) PL-HGD micelles could be converted into P-HGD nanoparticles to expose glucose ligands and partially release IMQ because of the desorption of mPEG-*b*-PLys in the TME. The converted P-HGD nanoparticles could target cancer cells and be uptaken by TAMs to induce DOX-mediated cancer cell apoptosis and IMQ-mediated polarization of TAMs towards the M1-like phenotype. Furthermore, the IMQ released in the TME could be absorbed by immature DCs, inducing their maturation and activation to in-turn activate cytotoxic T cells.

Based on this combined therapeutic strategy and drug interaction characteristics, a precise pH-responsive and TME-targeting micelle was designed to expose glucose ligands for enhanced uptake by cancer cells and the subsequent release of IMQ to induce TAM polarization and DC maturation, thereby achieving combined chemo- and immuno-therapy. In this study, two pH-responsive copolymers, poly(methyl methacrylate-*co*-glucosamine/histidine/doxorubicin) (P(MAA-*co*-GLU/HIS/DOX), abbreviated as M-HGD) and poly(propyl methacrylate-*co*-glucosamine/histidine/doxorubicin) (P(PAA-*co*-GLU/HIS/DOX), abbreviated as P-HGD) (Fig. 1A), with different pKa values of approximately 5.0 and 6.5 for MAA and PAA, respectively, were synthesized by free radical polymerization. The carboxylic groups of MAA and PAA were important pH-sensitive functional group. The NHS group of MAA-NHS was used to conjugate histidine, glucosamine and DOX. The chemotherapeutic drug, DOX, was conjugated by a pH-sensitive hydrazone bond on these copolymers. IMQ was then encapsulated with the M-HGD and P-HGD copolymers to form glucose-conjugating nanoparticles, which were then coated with a second polymer, methoxy-poly(ethylene glycol)-*block*-poly(l-lysine) (mPEG-*b*-PLys), synthesized by open-ring polymerization, through electrostatic interactions to form the micelles, hereafter denoted as ML-HGD and PL-HGD. In the TME, the carboxyl groups of PAA and imidazole groups of histidine on the PL-HGD could be protonated because of the pH changing from 7.4 to 6.5. Subsequently, mPEG-*b*-PLys could be desorbed from micelles to expose glucose ligands and partially release IMQ (Fig. 1B). The physically encapsulated IMQ was design for activating immune cells in TME, otherwise chemically conjugated DOX was plan to delivering to cancer cells. The exposed glucose ligands could significantly improve P-HGD nanoparticle delivery to cancer cells through endocytosis. The acidic surroundings of the endosomes induce the release of DOX from the copolymers through the hydrolysis of pH-sensitive hydrazone bonds for effective chemotherapy. Furthermore, P-HGD nanoparticles can also be internalized into TAMs and release the remaining IMQ in the phagosomes/phagolysosomes, thereby inducing the secretion of cytokines and the polarization of TAMs into M1-like macrophages for macrophage-mediated immunotherapy. Meanwhile, IMQ could be rapidly released into the TME because of the pH-sensitive characteristics of PL-HGD. A portion of the released IMQ could be absorbed by DCs in the TME. Afterward, the IMQ released in the TME could further mature the DCs and the cytotoxic T cells for cancer immunotherapy (Fig. 1C). Conversely, the PM-HGD performance lacked therapeutic efficacy on combinational therapy because the responsive site was in the late endosomes and secondary lysosomes instead of TME.

## 2 Experimental

### 2.1 Materials

Amino functionalized methoxyl polyethylene glycol, amino PEG (mPEG5000-amine) was purchased from Nanocs (Boston, Massachusetts, USA). MAA, 3-mercaptopropionic acid (3-MPA), and sodium bicarbonate (NaHCO_3_) were obtained from Acros organics (Geel, Belgium). The 2,2′-Azobisisobutyronitrile (AIBN) was purchased from UniRegion Bio-Tech Inc (Taiwan).; Glucosamine hydrochloride (GLU), histidine (HIS), *N*-hydroxysuccinimide (NHS), N,N′-dicyclohexyl-carbodiimide (DCC), *tert*-butyl carbazate, hydrobromic acid solution (33 ​wt % in acetic acid), and trifluoroacetic acid (TFA) were purchased from Sigma-Aldrich (St. Louis, Missouri, USA). The DOX hydrochloride was purchased from LC Laboratories (Woburn, Massachusetts, USA) and IMQ was purchased from TCI (Tokyo, Japan). 4-Dimethylaminopyridine (DMAP) was purchased from Alfa Aesar (Ward Hill, Massachusetts, USA); Acetonitrile (ACN), dichloromethane (DCM), dimethylformamide (DMF), dimethyl sulfoxide (DMSO), methanol (MeOH), ethanol, diethyl ether, and isopropyl alcohol (IPA) were purchased from ECHO (Miaoli, Taiwan). N6-Carbobenzoxy-l-lysine *N*-carboxyanhydride (NCA-lysine) was purchased from Carbosynth (Compton, Berkshire, UK.). PAA was purchased from Polymer Source (Dorval, Quebec, Canada). AIBN was recrystallized from MeOH before use. DCM, DMF, and DMSO were dried and distilled with calcium hydride before use.

### 2.2 Synthesis of *N*-(Methacryloyloxy) succinimide (MAA-NHS)

MAA (0.8 ​mL, 9.39 ​mmol), NHS (1.6230 ​g, 14.09 ​mmol), and DMAP (0.3445 ​g, 2.82 ​mmol) were dissolved in DCM (20 ​mL). A solution of DCC (3.8749 ​g, 18.78 ​mmol) in DCM (40 ​mL) was then added dropwise; the reaction was conducted at room temperature with stirring for 24 ​h. After the reaction was complete, acetic acid (0.4 ​mL, 6.99 ​mmol) was added to the mixture and incubated overnight at −20 ​°C. Reaction mixture was progressively filtered to remove 1,3-dicyclohexyl urea (DCU), extracted twice with saturated sodium bicarbonate solution and three times with deionized water. It was then evaporated in a rotary evaporator to obtain a white powder. The final product was recrystallized with IPA three times. ^1^H NMR (400 ​MHz, DMSO-*d*_6_, δ): 6.35 (s, 1H), 6.05 (s, 1H), 2.85 (s, 4H), 2.0 (s, 3H). FT-IR (KBr pellet): 2954 (O–H), 1761–1737 (C=O), 1631 (C=C), 1209–1087 (C–O).

### 2.3 Synthesis of P(PAA-co-NHS) and P(MAA-co-NHS) copolymers

P(PAA-*co*-NHS) and P(MAA-*co*-NHS) copolymers were synthesized by free radical polymerization [32]. Briefly, AIBN (7.6 ​mg, 0.046 ​mmol), PAA/MAA (190.2 ​mg, 1.667 mmol/54.5 ​mg, 0.621 ​mmol), MAA-NHS (169.6 ​mg, 0.896 ​mmol), and 3-MPA (24.6 ​mg, 0.232 ​mmol) were dissolved in a MeOH/DMSO co-solvent. The reactions were incubated for 24 ​h at 50 and 70 ​°C and then precipitated using cold diethyl ether to obtain P(PAA-*co*-NHS) and P(MAA-*co*-NHS), respectively. ^1^H NMR spectrum of P(PAA-*co*-NHS) (400 ​MHz, DMSO-*d*_6_, δ): 2.7–3.0 (br, 4H), 1.0–1.8 (br, 9H), 0.7–0.9 (br, 3H). FT-IR spectrum of P(PAA-*co*-NHS) (KBr pellet): 3446 (O–H), 2993–2962 (C–H), 1735–1676 (C=O), 1203–1064(C–O). ^1^H NMR spectrum of P(MAA-*co*-NHS) (400 ​MHz, DMSO-*d*_6_, δ): 2.7–3.0 (br, 4H), 1.6–2.1 (br, 2H), 0.7–1.5 (br, 3H). FT-IR spectrum of P(MAA-*co*-NHS) (KBr pellet): 3410 (O–H), 2997−2947 (C–H), 1726−1718 (C=O), 1215−1020 (C–O).

### 2.4 Synthesis of P-HGD and M-HGD copolymers

Either the P(PAA-*co*-NHS) or the P(MAA-*co*-NHS) copolymers were mixed with glucosamine hydrochloride in DMSO containing 10% TEA at 60 ​°C and incubated for 24 ​h. Then, histidine molecules were incubated with this mixture for 5 days for the reaction. After the reaction, excess amounts of *tert*-Butyl carbazate were added and incubated at 37 ​°C for 24 ​h. The mixture was then progressively precipitated using cold diethyl ether, reacted with TFA to remove the butyloxycarbonyl (boc)-protecting group for 2 ​h, and then conjugated with DOX at 37 ​°C for 24 ​h. The dark-red mixture was then placed in a dialysis bag (MWCO 1 ​K) against DMSO for 3 days and purified using a Sephadex LH-20 packing column to obtain P-HGD and M-HGD copolymers. The molecular weight and polydispersity index (PDI) were determined using gel permeation chromatography (GPC) system (LC-20AT, Shimadzu Co., Japan) with a Phenomenex Shodex OHpak ® SB-804 HQ column (10 ​μm, 300 ​mm ​× ​8.0 ​mm), and an RI detector (RID-10 ​A, Shimadzu Co., Japan) at 40 ​°C. The mobile phase was DMF containing 50 ​mM lithium bromide with a flow rate of 1 ​mL/min ^1^H NMR of P-HGD (400 ​MHz, DMSO-*d*_6_, δ): 7.3–8.7 (br, 3H), 6.5–7.3 (br, 1H), 5.1–5.8 (br, 1H), 1.5–2.3 (br, 2H), 0.9–1.5 (br, 7H), 0.2–0.9 (m, 3H). FT-IR of P-HGD (KBr pellet): 3443 (O–H), 2976−2877 (C–H), 1720−1666 (C=O), 1192−1016 (C–O). ^1^H NMR of M-HGD (400 ​MHz, DMSO-*d*_6_, δ): 7.1–8.8 (br, 3H), 6.6–7.1 (br, 1H), 5.2–5.6 (br, 1H), 1.5–2.2 (br, 2H), 0.3–1.5 (br, 3H). FT-IR of M-HGD (KBr pellet): 3441 (O–H), 2999−2949 (C–H), 1716−1676 (C=O), 1188−1018 (C–O).

### 2.5 Synthesis of mPEG-b-PLys

mPEG5000-amine was used as a macroinitiator for the ring-opening polymerization. Fixed amounts of mPEG5000-amine and NCA-lysine were dissolved DMF (50 ​mL) under nitrogen. The reaction was conducted at 35 ​°C for 2 days. The mixture was then progressively precipitated using ethanol, reacted with 33% HBr in acetic acid at room temperature to remove carboxybenzyl protecting group, and precipitated using cold diethyl ether to obtain mPEG-*b*-PLys. ^1^H NMR (400 ​MHz, D2O, δ): 4.1–4.2 (br, 26H), 3.4–3.7 (br, 455H), 3.2–3.3 (br, 3H), 2.8–3.0 (br, 27H), 1.1–1.8 (br, 162H). FT-IR (KBr pellet): 3771 (N–H), 2885 (C–H), 1656−1627 (C=O), 1118 (C–O).

### 2.6 Preparation and basic characterization of micelles

Fifteen mg of the M-HGD or the P-HGD copolymers were dissolved in 1.5 ​mL of DMSO and mixed with 2 ​mg of IMQ in 4 ​mL of MeOH. Core nanoparticles were then prepared using a solvent exchange process with a dialysis bag (MWCO 6–8 ​K) against deionized water. The water was changed every 12 ​h. After 3 days, the core nanoparticle solution was passed through a Sephadex G-50 packing column using phosphate buffer solution (PBS) with a pH of 7.4 as the elution solvent to remove free IMQ. The core nanoparticle solution was then mixed with 15 ​mg of mPEG-*b*-PLys and stirred for 24 ​h at room temperature to form micelles. Finally, the solution was progressively filtered through a 0.8-μm filter to remove precipitates and then centrifuged using an Amicon ultra centrifugal filter (30 ​K) to remove free mPEG-*b*-PLys. Particle size and polydispersity index (PDI) were measured for the micelles using dynamic light scattering (DLS, Zetasizer Nano ZS90, Malvern Panalytical, UK). The maximum absorption and emission spectrum was determined using UV/Vis spectrophotometer (Ultrospec 9000pc, Biochrom, United Kingdom) and plate reader (Infinite M200 Pro, TECAN, Switzerland), respectively. The morphologies of the micelles were observed by transmission electron microscopy (TEM, JEM-2000EXII, JEOL, Japan) at an accelerating voltage of 120 ​kV. In order to determine the stability of the micelles, sterile micelles were stored at 4 ​°C. At different time intervals, 10 ​μL of concentrated micelle solution was diluted with 1 ​mL of PBS; the size change was monitored for 2 months using DLS. In order to determine the pH-responsiveness of the micelles, 10 ​μL of the concentrated micelle solution was diluted with 1 ​mL of PBS at different pH levels (pH of 7.4, 6.5, and 5.0) and incubated at 37 ​°C. The hydrodynamic diameters of the micelles were measured at different time points using DLS. The accurate drug loading of IMQ was measured using a HPLC (Shimadzu Co., Japan) with an Inspire™ C18 column (5 ​μm, 250 ​mm ​× ​4.6 ​mm), and an UV–Vis detector (SPD-10 ​A, Shimadzu Co., Japan) at 40 ​°C. The mobile phase comprised 70% sodium acetate (pH ​= ​4) and 30% ACN with a flow rate of 1 ​mL/min. The quantitative analysis of the IMQ was performed at 244 ​nm. In contrast, the drug loading of DOX was measured by UV/Vis spectrophotometer at 480 ​nm. Drug loading measurement was calculated according to the formula as following:

Drugloading=(weightofloadeddrug/totalweightofnanoparticles)×100%

### 2.7 IMQ- and DOX-releasing behaviors of micelles

In order to understand the IMQ- and DOX-releasing behaviors of the micelles, 1 ​mL of the micelle solution was transferred into a dialysis bag (MWCO 6–8 ​K) and immersed in 3 ​mL of PBS at different pH levels and incubated at 37 ​°C. At different time intervals, 3 ​mL of sample was collected, filtered through a 0.45-μm PVDF filter. The analyzed method was modified from HPLC procedure as mentioned before. HPLC equipped with UV–Vis detector (SPD 10 ​A, Shimadzu Co., Japan)/fluorescence detector (RF-10 AXL, Shimadzu Co., Japan). DOX was measured by detecting the fluorescence at an excitation and emission wavelength of 480 and 570 ​nm.

### 2.8 Cytotoxicity evaluation

4T1 (8 ​× ​10^4^ ​cells/mL), RAW 264.7 (2 ​× ​10^5^ ​cells/mL), and L929 ​cells (1 ​× ​10^5^ ​cells/mL) were seeded on individual 96-well plates (each well contained 0.1 ​mL of cell suspension). After 12 ​h of incubation, various concentrations of free DOX, IMQ, DOX/IMQ, and micelles were added to the wells for 24 ​h. After removing drug/micelle-containing medium, the MTT assay was performed to determine the viability of each cell sample using an ELISA reader (Infinite M200 Pro, TECAN, Switzerland).

### 2.9 Cellular uptake of micelles

4T1 cells (5 ​× ​10^5^ ​cells/each well) were seeded on 6-well plates. After 12 ​h of incubation, cells were pretreated with 100 ​mg/mL of glucose for 1 ​h. Subsequently, either free DOX or micelles (at a DOX concentration of 1 ​μg/mL) along with 100 ​mg/mL of glucose were added to each well. After 3 ​h of co-incubation, the cells were harvested and washed with cold PBS. The intracellular fluorescence intensity for DOX was detected using flow cytometry (FACSCalibur, BD Biosciences, USA). For intracellular fluorescence observation of free DOX and micelles, 4T1 cells were seed in 18 ​× ​18 ​mm cover glass. After 12 ​h, the medium was changed with various pH PBS containing 10% FBS and various formulated DOX. After 2 ​h, 1 ​μL of 50 ​μM LysoTracker Deep Red (Thermo Fisher; catalog: L12492) was added and treated for further 1 ​h. The cells were washed twice with cold PBS, fixed with 10% formalin and then sealed with DAPI-contained mounting gel. The intracellular distribution of different formulated DOX was observed by confocal fluorescence microscopy (LSM880, Zeiss, Germany).

### 2.10 Activation of RAW 264.7 cells by micelles

RAW 264.7 ​cells were seeded at a density of 10^6^ ​cells/well into 6-well plates. After 12 ​h, the culture medium was replaced with 2 ​mL of culture medium containing either free IMQ or micelles and incubated for another 6 ​h. Then, the cell culture supernatant was collected for TNF-α (eBioscience, catalog: 88-7324-88) and IL-6 (eBioscience, catalog: 88-7064-88) analysis using ELISA.

### 2.11 Hemocompatibility study

The method was modified from that used in a previous study [33]. In brief, fresh whole blood was collected from the mice in a heparin-containing tube. To remove the serum, the whole blood sample was centrifuged at 200 ​g for 5 ​min. The precipitated red blood cells (RBCs) were washed with sterile PBS five times and diluted with PBS after the last wash. A total of 0.2 ​mL of diluted RBC was mixed separately with either 0.8 ​mL of sterile PBS (negative control; NC), purified water (positive control; PC), free DOX, and micelles. Afterward, the mixture was incubated for 3 ​h. The supernatant was obtained by centrifugation at 2000 RPM for 10 ​min. The absorbance of hemoglobin in the supernatant was measured at 570 ​nm, and the reference was set at 620 ​nm.

The hemolysis percentage was calculated according to the following equation:

$$Hemolysis\left(\%\right)=\frac{{Sample\;Abs}_{total}-{Sample\;Abs}_{\;dox\;or\;micelles}-NC\;Abs}{PC\;Abs-NC\;Abs}X\;100\%$$

### 2.12 Biodistribution of micelles in an orthotopic implant animal model

Female BALB/c mice (4–6 weeks old) were purchased from the National Laboratory Animal Center (Taipei, Taiwan). All the animals were managed and experiments were performed in accordance with the Guidance on the Usage and Care of Laboratory Animals, approved by the institutional animal care and use committee (IACUC) of National Yang-Ming University. The 4T1 cells (1 ​× ​10^6^ ​cells in 100 ​μL of PBS) were translocated into the center of the right mammary fat pad of each mouse. Tumor volume was calculated using the following formula: tumor volume (mm^3^) ​= ​longest side ​× ​shortest side^2^/2.4T1 tumor-bearing BALB/c mice with tumor volumes of approximately 100–250 ​mm^3^ were administrated with Cy5.5-labeled micelles via tail vein injection. The organs and tumors were harvested and analyzed using in vivo imaging systems (PhotonIMAGER Optima, Biospace Lab, France) 24 ​h later. 4T1 tumor-bearing BALB/c mice with tumor volumes of approximately 100–250 ​mm^3^ were intravenously injected with free DOX, ML-HGD, and PL-HGD at a dose equivalent to 8 ​mg/kg of DOX. After 24 ​h, the mice were sacrificed and the tumor, liver, kidney, spleen, and lung were collected and embedded in Tissue-Tek® optimum cutting temperature (O.C.T.) solution with a liquid nitrogen bath. These tissues were cut to have a 10 ​μm thickness using a Cryostat Microtome (CM3050S, Leica, Germany). Next, the sliced tissue samples were sealed with mounting medium containing DAPI; the fluorescence intensities of DOX and DAPI were observed by confocal fluorescence microscopy (LSM880, Zeiss, Germany). For fluorescence image observation, the excitation/emission wavelength for DOX and DAPI was 480/595 ​nm and 405/450 ​nm, respectively.

### 2.13 Tumor inhibition in an orthotopic implant animal model

The 4T1 cells (1 ​× ​10^6^ ​cells in 100 ​μL of PBS) were translocated into the center of the right mammary fat pad of each mouse. After 1 week of tumor implantation, mice with an average tumor volume of 50–200 ​mm^3^ were randomly assigned to 6 groups (each group contained 6 mice): PBS, DOX, IMQ, DOX ​+ ​IMQ, PL-HGD, and 2-fold PL-HGD. The tumor-bearing mice were intravenously injected with 8 ​mg/kg of DOX and/or 25 ​μg/kg of IMQ (or PL-HGD at equivalent DOX and IMQ doses) four times, once every 3 days. Tumor volume and body weight were measured every 2 days. Mice were sacrificed on the 12th day from the first intravenous injection and whole blood samples were collected. Red blood cells (RBCs) and white blood cells (WBCs) were analyzed using a hematology analyzer (XT-1800iv, Sysmex, Japan). The remaining whole blood samples were centrifuged at 2000×*g* for 10 ​min to obtain plasma components for the evaluation of hepatic and renal function indexes such as glutamic pyruvic transaminase (GPT) and blood urea nitrogen (BUN) using an automated clinical chemistry analyzer (Fuji Dri-Chem 4000i, Fujifilm, Japan).

### 2.14 Immune status evaluation

In order to evaluate the activation of DCs and polarization of macrophages, the right inguinal lymph nodes and tumors were isolated and dissociated into single cells by incubating with Accumax for 1 ​h. The cell suspension was passed through a 40-μm cell strainer to remove tissue masses and stained with PE Anti-Mouse CD86 (eBioscience, catalog: 17-0862-82), FITC Anti-Mouse CD80 (eBioscience, catalog: 11-0801-82), FITC Anti-Mouse CD11c (eBioscience, catalog: 11-0114-82), Alexa Fluor® 647 Anti-Mouse CD206 (Bio-Rad, catalog: MCA2235A647), FITC Anti-Mouse CD86 (Invitrogen, catalog: 11-0862-85), PE Anti-Mouse F4/80 (Elabscience, catalog: E-AB-F0995D), APC Anti-Mouse CD3 (BioLegend, catalog: 100,236) and FITC Anti-Mouse CD8 (Invitrogen, catalog: 11-0862-85). After 30 ​min, the cell suspension was centrifuged at 2000 ​rpm to collect the cells and then washed twice with cold PBS. The fluorescent marker was detected using a flow cytometer (Coulter CytoFLEX, Beckman coulter, USA). In order to observe CD3^+^ and CD8^+^ T cells in tumor tissues, the tumors were sequentially fixed with 3.7% paraformaldehyde, embed in parafilm, cut into 5-μm thick slices, and incubated with the primary antibody against anti-mouse CD3 (eBioscience, catalog: 14-0032-82), CD8 (eBioscience, catalog: 14-0808-82), and TNF-α (GeneTex catalog: GTX110520). Goat anti-mouse IgG and HRP-linked antibodies were the secondary antibodies used to bind the primary antibodies. Immunohistochemistry (IHC) images were observed using phase contrast microscopy (Invitrogen™ EVOS™ XL Imaging System, Thermo Fisher, USA). In order to evaluate the expression of iNOS, the tumor tissues were embedded in O.C.T. solution and frozen at −20 ​°C. These tissues were cut into 10-μm thick slices using a Cryostat Microtome (CM3050S, Leica, Germany). Next, the sliced tissue sample was blocked with PBS buffer containing 1% BSA and 0.1% Tween 20. After 30 ​min, the tissue slice was stained with diluted iNOS antibody (Miltenyi Biotec, catalog: 130-116-357) at 4 ​°C overnight. The sample was sealed with mounting medium containing DAPI and observed by confocal fluorescence microscopy (LSM880, Zeiss, Germany).

### 2.15 Statistical analysis

All experimental data were presented as the mean ​± ​standard deviation (SD) from at least three independent experiments. The p-values were calculated by one-way ANOVA. A p-value lower than 0.05 indicated a statistically significant difference.

## 3 Results and discussion

### 3.1 Synthesis and characterization of copolymers and micelles

The number of carbons on acrylic acid monomer has been reported to affect the pKa values of acrylate polymers [34]. The pKa values of PAA and MAA have been reported to be similar to the extracellular and intracellular pH values in tumors [35], respectively; therefore, drug delivery systems comprising PAA and MAA can control the release of the drug both outside and inside the cancer cells. This enables the evaluation of the difference between the therapeutic efficacy of drug delivery systems on the TME and that on intracellular endosomes. Procedures for the synthesis of P-HGD and M-HGD copolymers have been illustrated in Fig. S1. The synthesized materials were characterized using ^1^H NMR and FT-IR to identify their structure and purity (Fig. S2–S6 and Table S1–S2). The average molecular weights and PDI of the copolymers were measured by GPC (Fig. S7) and summarized in Tables S1 and S2. The experimental results indicate that the molecular weight and polydispersity index (PDI) of the M-HGD and P-HGD copolymers were similar (Fig. S7B). From the ^1^H NMR analysis, the DOX contents (molar percentages) in the P-HGD and M-HGD copolymers were 23 and 19%, respectively, which were similar to those confirmed by UV–Vis spectrum (20.7% and 19.1%). In the result of titration, M-HGD copolymers exhibited two pKa values of approximately 4.5 and 6.8 contributed by the carboxylic acids of the methylacrylic acid and imidazole groups of histidine, respectively. In contrast, P-HGD copolymers showed a pKa value of 6.8 from histidine and propylacrylic acid (Fig. S8). The mPEG-*b*-PLys copolymers were synthesized by ring-opening polymerization, after which the carbobenzoxy protecting groups were removed to expose their positive charges. The structure of the copolymer was confirmed by analyzing the ^1^H NMR and FT-IR spectra (Fig. S9). The mPEG-*b*-PLys copolymers contained 27 Lys residues, as the repeating units.

**Fig. 2:**
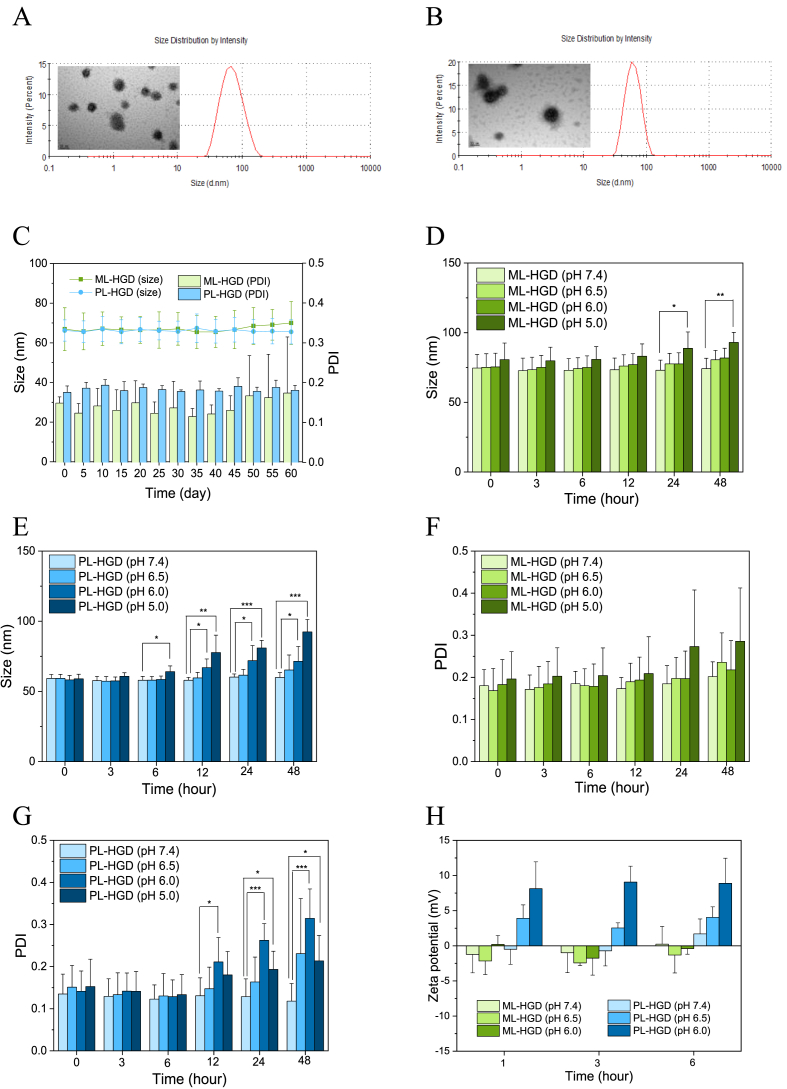
The properties of ML-HGD and PL-HGD micelles. The size distribution and TEM morphology of (A) ML-HGD and (B) PL-HGD. (C) The long-term stability of ML-HGD and PL-HGD at 4 ​°C for 2 months. The data have been expressed as mean ​± ​SD from five independent experiments. The changes in (D and E) particle size, (F and G) PDI, and (H) zeta-potential for ML-HGD and PL-HGD at various pH levels. The data have been expressed as mean ​± ​SD from at least three independent experiments.

Copolymers become hydrophobic after conjugating with DOX; therefore, a solvent exchange process involving dialysis was used to prepare the nanoparticles and encapsulate a hydrophobic drug, IMQ. Core nanoparticles exhibited negative charges of approximately −30 mV because the pKa values of PAA and MAA were below the pH level of 7.4 (Table S3). The average particle size and PDI of the core nanoparticles (M-HGD and P-HGD) were larger than 130 ​nm and 0.2, respectively. The accumulation of nanoparticles with high negative charges in the liver has been shown to be significantly higher than those of slightly negative and neutral nanoparticles [36]; therefore, mPEG-*b*-PLys copolymers were used to shield the surface charges through electrostatic interactions between PLys and the MAA or PAA to form micelles. The DLS analysis results and drug loading summarized in Table 1 show that the average particle size and PDI of both micelles were decreased to approximately 60 ​nm and 0.15, respectively, because the electrostatic interactions compressed the core structures. The zeta-potentials for ML-HGD and PL-HGD also changed to 1.0 and 3.5 mv, respectively, indicating that the mPEG molecules were outside the micelles. Both drug loading of DOX and IMQ were similar in ML-HGD and PL-HGD micelles. The morphologies of micelles were observed by TEM, which revealed that both micelles had spherical structures and their sizes were similar to those measured by DLS (Fig. 2A and B; Fig. S10 and S11). The UV–Vis analysis results indicate that the absorption peak of DOX after conjugation on polymers shifted from 481 to 507 ​nm (Fig. S12A), indicating the formation of a hydrazone bond [37]. In addition, the maximum absorbance and fluorescence intensity of micelles dramatically increased when micelles were suspended in DMSO (Fig. S12B). The quenching effect was observed because of the spatial proximity of the DOX molecules. The long-term stability for ML-HGD and PL-HGD were monitored for 2 months at 4 ​°C. For PL-HGD, the average particle size and PDI were maintained at around 60 ​nm and 0.2, respectively. Although the size of ML-HGD did not change, the PDI slightly increased and showed a high deviation after 50 days (Fig. 2C), suggesting that it was less stable than PL-HGD in PBS. ML-HGD and PL-HGD contained pH-responsive materials such as MAA, PAA, and histidine molecules that allowed them to change their structures at different pH levels. The pH-triggered size changes were evaluated using various pH buffers to mimic blood circulation (pH 7.4), TME (pH 6.5 and 6.8), and intracellular endosomes/secondary lysosomes (pH 5.0). Both ML-HGD and PL-HGD exhibited stability at a pH of 7.4 in PBS (Fig. 2D–G). However, the particle size or PDI were significantly different at pH levels of 6.5, 6.0, and 5.0 for PL-HGD at 12 ​h (Fig. 2E,G). As time increased, the particle sizes and PDI notably increased at both pH 6.0 and pH 5.0 compared to those at pH 7.4. For ML-HGD, particle size and PDI were only varied at a pH of 5.0 (Fig. 2D,F). Form the results of TEM, ML-HGD exhibited integral structures when the pH changed from 7.4 to 6.0. However, an increased size of ML-HGD was observed at pH 5.0 due to structural deformation. In contrast to ML-HGD, PL-HGD exhibited a swollen and increased size in a mildly acidic environment, which was consistent with the results obtained with DLS measurement (Fig. S13). The zeta potential of PL-HGD was largely increased after 1 ​h at a pH of 6.0 in PBS, whereas ML-HGD retained their zeta-potentials even after 6 ​h at a pH of 6.0 (Fig. 2H). These experimental results indicate that PL-HGD could be protonated and desorbed from the mPEG-*b*-PLys at the TME (pH 6.5–6.8) to expose its positively charged histidine molecules and targeting ligands of glucosamine molecules. In contrast, MAA is protonated at a pH of 5.0; therefore, ML-HGD responded to pH changes not at the TME, but in intracellular endosomes/secondary lysosomes.

**Table 1:** Characterization of ML-HGD and PL-HGD micelles.

	In feed			Characteristicsa)			Drug loading	
	M-HGD (mg)	P-HGD (mg)	IMQ (mg)	Size (nm)	PDI	Zeta(mv)	DOXb) (%)	IMQc) (%)
ML-HGD	15	–	2	65.3 ​± ​11.8	0.164 ​± ​0.070	1.0 ​± ​2.6	8.1 ​± ​2.0	0.004 ​± ​0.001
PL-HGD	–	15	2	61.8 ​± ​4.4	0.149 ​± ​0.041	3.5 ​± ​1.4	8.0 ​± ​0.2	0.005 ​± ​0.002

a) Characteristics of micelles were determined by DLS.

b) The DOX concentration was quantitated using UV–Vis spectroscopy.

c) The IMQ concentration was determined by HPLC. The data was expressed as mean ​± ​SD of five independent experiments.

### 3.2 Drug release and targeting abilities of micelles

**Fig. 3:**
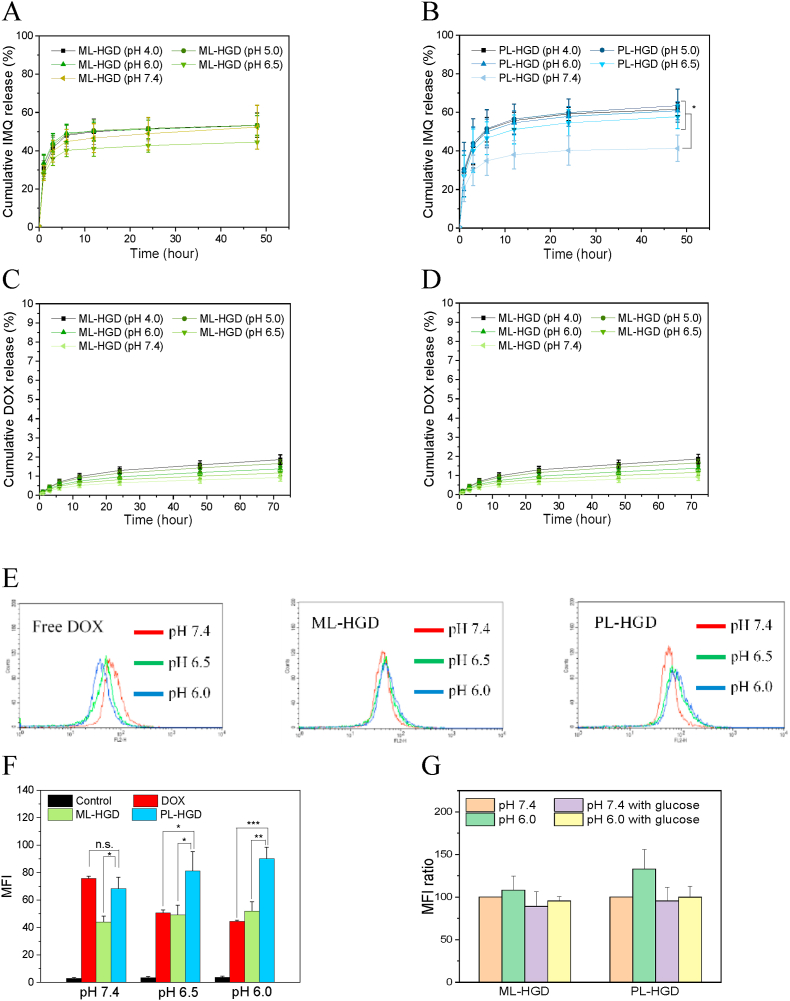
Drug release and cancer cell targeting abilities of ML-HGD and PL-HGD. The cumulative IMQ- and DOX-release behavior of (A and C) ML-HGD and (B and D) PL-HGD at various pH levels. The data have been expressed as mean ​± ​SD from at least three independent experiments. (E) The histogram of flow cytometry analysis of 4T1 cells treated with free DOX, PM-HGD, and PL-HGD at various pH levels for 3 ​h. (F) Quantitative analysis of flow cytometry for 4T1 cells treated with drugs. (G) The MFI ratio of 4T1 cells treated by ML-HGD and PL-HGD with and without glucose (100 ​mg/mL) pre-treatment. The data have been expressed as mean ​± ​SD from three independent experiments. (n.s., nonsignificant; ∗P ​< ​0.05; ∗∗P ​< ​0.01; and ∗∗∗P ​< ​0.001.)

ML-HGD exhibit less pH-triggered IMQ release behavior (Fig. 3A). In contrast, the cumulative release percentage of IMQ from PL-HGD was significantly increased below a pH of 6.5 compared to that at a pH of 7.4 because the protonation of PAA and histidine makes the PL-HGD swollen (Fig. 3B). The experimental results indicate that PL-HGD could release IMQ at the TME for the activation of DCs and TAMs. The cumulative release percentage of IMQ at pH 6.5 was increased by 20% compared to pH 7.4, exhibiting statistically significant differences. This study has revealed that only 1 ​μg/mL of IMQ could cause proinflammatory cytokine secretion of bone-marrow-derived dendritic cells [38]. Therefore, the IMQ released from micelles at pH 6.5 was sufficient to induce the activation of DCs. On the other hand, DOX was conjugated onto the side chain of copolymers through hydrazone bonds; therefore, both ML-HGD and PL-HGD released low amounts of DOX in neutral and acidic surroundings (pH ​< ​7) (Fig. 3C and D). The M-HGD and P-HGD copolymers were observed to aggregate in DMSO (Fig. S14), suggesting that the hydrophobicity of the copolymers was remarkably increased after conjugating with glucosamine, histidine, and DOX. This result suggests that the hydrophobicity of the copolymers was remarkably increased after conjugating with glucosamine, histidine, and DOX. Furthermore, drug release behavior has been reported to be associated with the hydrophobicity of nanoparticles [39]; therefore, it is challenging to hydrolyze the hydrazone bonds and release the DOX from the tight structure of core nanoparticles. However, the changes in the sizes of PL-HGD were more noticeable than those of ML-HGD, leading to a faster cumulative release of DOX from PL-HGD than from ML-HGD as pH decreases. Previous studies have shown that acid hydrolases in the secondary lysosomes might be helpful in facilitating hydrazone bond cleavage between the DOX and polymers [40,41]. Therefore, the relatively fast release of DOX from PL-HGD in cancer cells was expected.

**Fig. 4:**
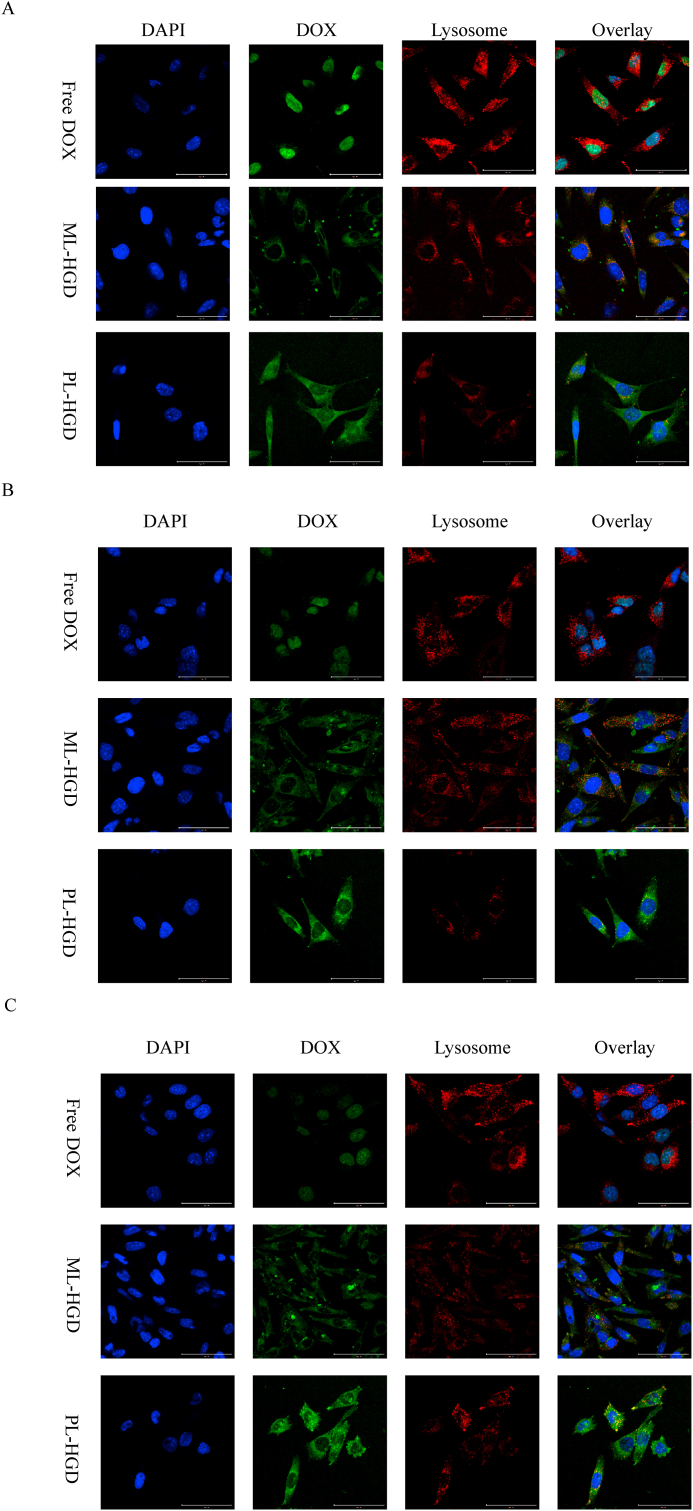
The confocal fluorescence images of 4T1 cell incubated with free DOX, ML-HGD and PL-HGD at (A) pH 7.4, (B) pH 6.5 and (C) pH 6.0 ​PBS containing 10% FBS for 3 ​h. The scale bar is 50 ​μm. Blue fluorescence represents the cell nucleus stained with DAPI. Green fluorescence represents the DOX. Red fluorescence represents the lysosome stained with LysoTracker™ Deep Red.

Glucose Transporter 1 (GLUT1) is overexpressed in various mammary carcinoma cells such as 4T1, MCF-7, and MDA-MB-231 [42,43]; therefore, glucose has been used as an active ligand of nanoparticles to target cancer cells [26,44]. An acidic TME is known to be closely associated with tumor chemosensitivity [45,46]. In a mildly acidic environment (such as TME), DOX, a common chemotherapeutic drug with a low base pKa value, becomes charged and increases the polarity leading to higher hydrophilicity; therefore, DOX can hardly pass through the cytoplasmic membranes, which reduces its cytotoxicity and therapeutic efficacy [47]. Here, in Fig. 3E and F, the cellular uptake of DOX significantly decreased (around 50% of the DOX mean fluorescence intensity (MFI)) as the pH level changed from 7.4 to 6.0. In contrast, the MFI for ML-HGD was almost the same in neutral and acidic surroundings. The cellular uptake of ML-HGD was lower than that of PL-HGD, probably because of the DOX quenching effect in nanoparticles and lower cumulative release rates in acidic surroundings. Moreover, MFIs of the PL-HGD at pH levels of 6.5 and 6.0 were 19.1 and 32.4%, respectively, which were higher than that at a pH of 7.4. The cellular uptake of PL-HGD at pH 6.0 was 1.32-fold higher than that at pH 7.4. After pretreatment with 100 ​mg/mL glucose at pH 6.0, the cellular uptake of PL-HGD decreased to 109%, indicating that the positive charges only promoted approximately 9% of cellular uptake (Fig. 3G). The MFI for PL-HGD was 2.5-fold higher than that for free DOX at pH 6.0, suggesting that PL-HGD could increase the intracellular accumulation of DOX because glucose ligands get exposed at low pH levels. In addition, the fluorescent images indicate that free DOX accumulated in the nucleus but separated with cytoplasm. The fluorescence intensity of the free DOX decreased with decreasing pH. In contrast, the DOX signal released from the ML-HGD was co-localized with the LysoTracker and slightly increased as pH decreased. However, the DOX released from the PL-HGD occupied both the nucleus and cytoplasm. The fluorescent intensity for the PL-HGD noticeably increased as the pH decreased and was much higher than that for the ML-HGD, indicating that the PL-HGD had targeted and rapid drug release abilities at low pH (Fig. 4). However, the MFI for PL-HGD at a pH of 6.0 was dramatically decreased in the glucose-pretreated competition test, suggesting the inhibition of GLUT-1-mediated internalization of PL-HGD. These cell uptake experiment results indicate that PL-HGD could expose the targeting ligand in the TME and improve DOX accumulation in cancer cells to overcome the low penetration of DOX in acidic surroundings.

### 3.3 Cell cytotoxicity of micelles

**Fig. 5:**
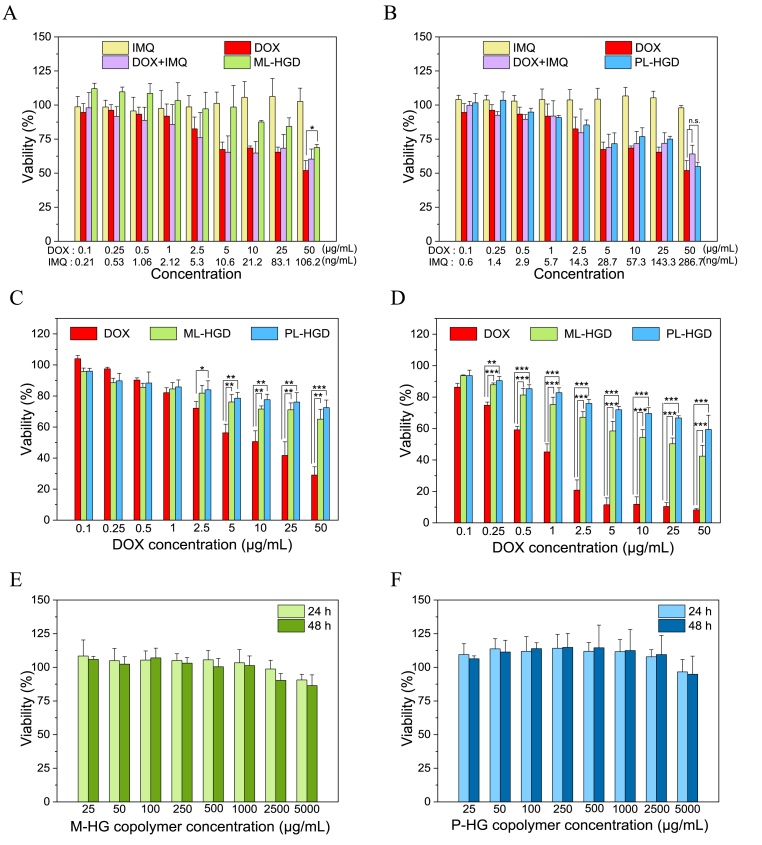
Cytotoxicities of ML-HGD and PL-HGD on 4T1 cancer cells and L929 fibroblast cells. The cell viability of 4T1 cells treated with (A) ML-HGD and (B) PL-HGD for 24 ​h. The L929 ​cell viability after treatment with ML-HGD or PL-HGD for (C) 24 ​h and (D) 48 ​h. The cytotoxic effects of (E) M-HG copolymers (without conjugating DOX) and (F) P-HG copolymers (without conjugating DOX) on 4T1 cells. The data have been expressed as mean ​± ​SD from at least three independent experiments. (n.s., nonsignificant; ∗P ​< ​0.05; ∗∗P ​< ​0.01; and ∗∗∗P ​< ​0.001).

In order to understand the cytotoxicity of the micelles on cancer and normal cells, 4T1 and L929 ​cells were co-incubated with DOX, IMQ, DOX combined with IMQ, and micelles at various concentrations. The experimental results reveal that IMQ was non-toxic to 4T1 cells because the concentrations of IMQ were too low to damage cells (Fig. 5A and B). Furthermore, DOX combined with IMQ exhibited similar cytotoxicity as DOX alone, indicating a non-synergistic effect at these concentrations. The cytotoxicity of PL-HGD treatment on 4T1 cells was similar to those of free DOX and DOX combined with IMQ, indicating that the DOX released from PL-HGD efficiently damages 4T1 cells. In contrast, the cytotoxicity of ML-HGD was lower than those of free DOX and PL-HGD because of lower DOX releasing ability. On the other hand, the cytotoxicity of ML-HGD and PL-HGD on L929 ​cells were much lower than that of DOX (Fig. 5C and D), suggesting that free DOX was toxic to normal cells, whereas micelles were not. In order to demonstrate that the cytotoxic effects of micelles were induced by DOX, the cytotoxicities of copolymers, without DOX conjugation, on 4T1 cells was evaluated. The M-HG and P-HG copolymers without DOX conjugation did not exhibit cytotoxicity even at a concentration of 1000 ​μg/mL (Fig. 5E and F). These cytotoxicity results indicate that micelles exhibited effective cancer cell cytotoxicity even though the release of DOX from micelles was slow.

**Fig. 6:**
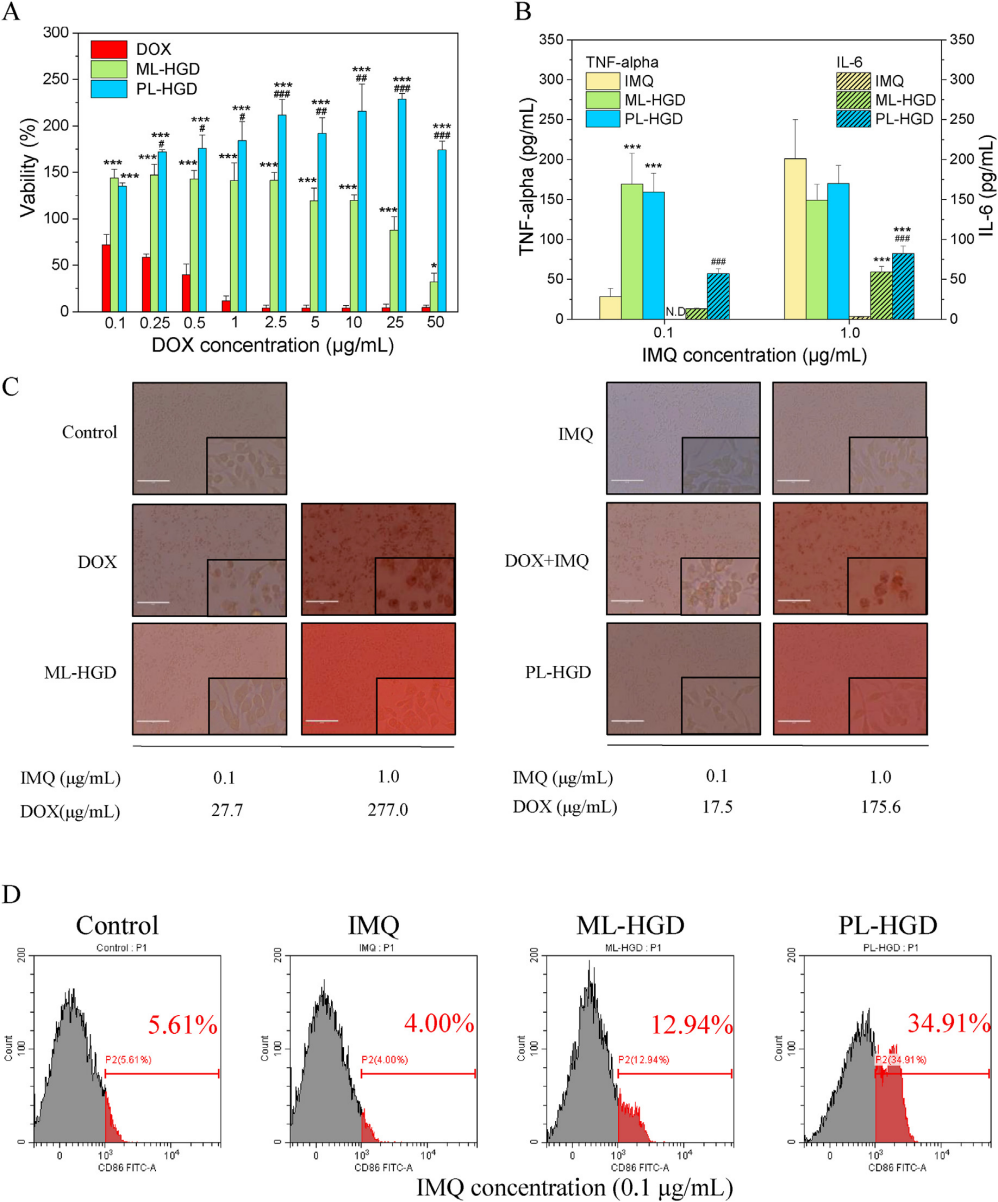
The effects of ML-HGD and PL-HGD on RAW 264.7 ​cells. (A) The viability of cells after treatments with DOX, ML-HGD, and PL-HGD for 24 ​h. (B) Concentrations of TNF-α and IL-6, (C) cell morphologies and (D) the percentage of CD86-positive cells after treatments with IMQ, ML-HGD, and PL-HGD for 6 ​h (∗P ​< ​0.05, ∗∗P ​< ​0.01 and ∗∗∗P ​< ​0.001 as compared with free DOX group; #P ​< ​0.05, ##P ​< ​0.01 and ###P ​< ​0.001 as compared with ML-HGD group).

Cytotoxic effects of free DOX and micelles on RAW 264.7 ​cells were also evaluated by the MTT assay. After 24 ​h of incubation, free DOX exhibited a high cytotoxicity to RAW 264.7 ​cells (Fig. 6A). In contrast, both ML-HGD and PL-HGD induced the proliferation of RAW 264.7 ​cells at low DOX concentrations because of the regulatory effects of IMQ [48]. However, the cell viability dramatically decreased at high DOX concentrations for ML-HGD treatment because cytotoxic effect of DOX was stronger than the proliferation effect induced by IMQ. PL-HGD induced higher cell proliferation than ML-HGD because of the high release rate of IMQ at low pH levels. These cytotoxicity results showed that DOX is highly cytotoxic to cancer cells (4T1), fibroblasts (L929), and macrophages (RAW 264.7). In contrast, PL-HGD caused 4T1 cell death and were less cytotoxicity to L929 and RAW 264.7 ​cells.

TLR7 is localized in phagosomes and can be used to sense foreign single-stranded RNA [49]; therefore, its activation can induce three downstream signaling pathways including mitogen-activated protein kinases (MAPKs), interferon regulatory factors (IRFs), and nuclear factor kappa–light-chain-enhancer of activated B cells (NF-κB). Eventually, proinflammatory cytokines including IL-6, IL-12, IL-18, and TNF-α can be produced by M1-like macrophages [50]. Our cytokine secretion results indicated that both ML-HGD and PL-HGD can significantly induce TNF-α and IL-6 secretion at low (0.1 ​μg/mL) and high (1.0 ​μg/mL) doses of IMQ; whereas, free IMQ only elevated the level of TNF-α (Fig. 6B). The cytokine secretion performance of PL-HGD was better than that of ML-HGD. The morphologies of RAW 264.7 ​cells were observed after treatment with free drugs and micelles. RAW 264.7 ​cells treated with DOX or DOX combined with IMQ had a spherical shape (dead cells) (Fig. 6C); whereas, RAW 264.7 ​cells had a spindle-shaped morphology (M1 type) after treatment with IMQ alone or micelles. CD86, a M1 type macrophage marker, was also evaluated at low (0.1 ​μg/mL) doses of IMQ and micelles. The expression level of CD86 on RAW 264.7 ​cells treated with PL-HGD was significantly increased among treating groups (Fig. 6D). These experimental results suggest that PL-HGD could polarize macrophages to M1 phenotype as compared to free IMQ and ML-HGD.

### 3.4 Functions of micelles in 4T1 orthotopic tumor-bearing mice

**Fig. 7:**
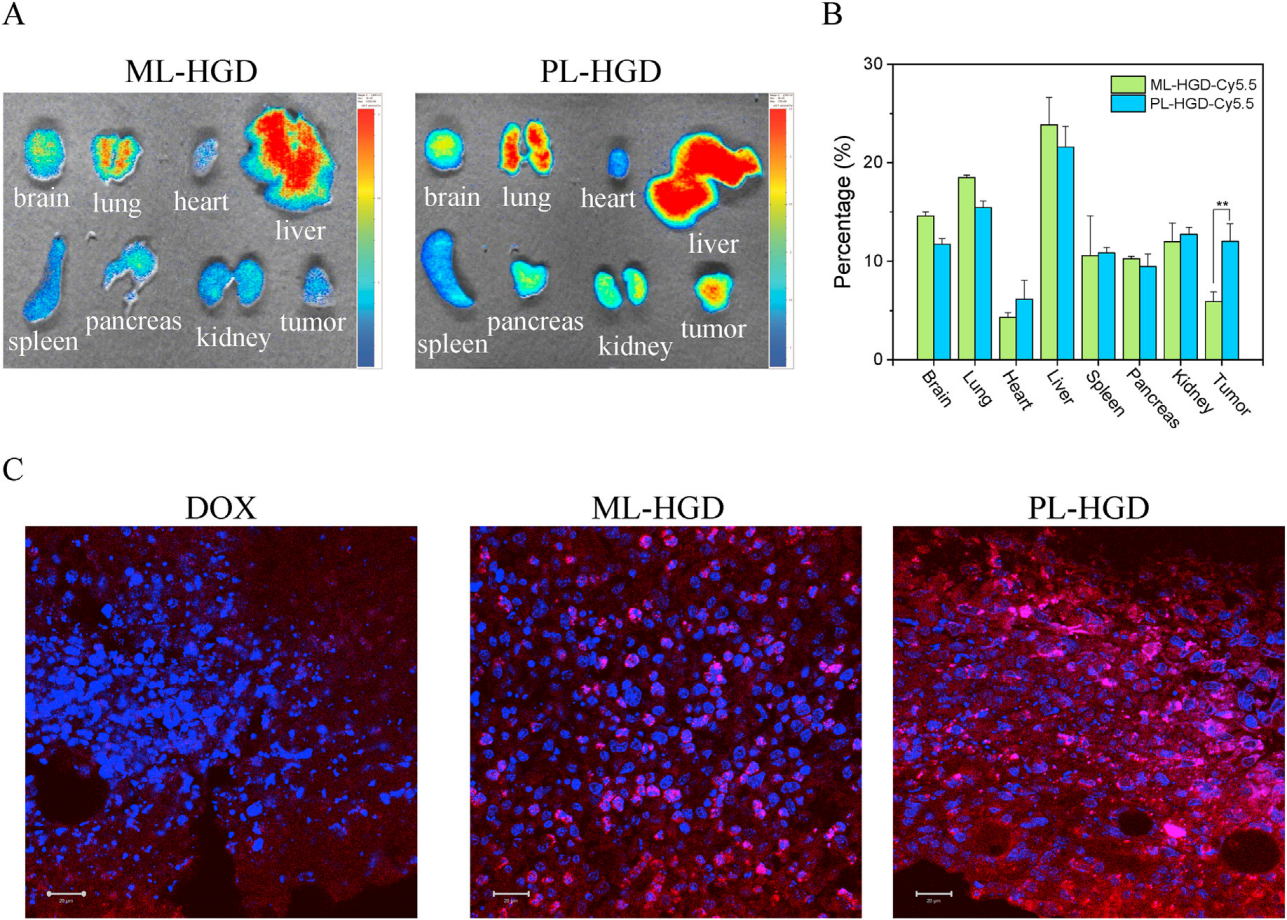
Tumor and organ distribution of ML-HGD and PL-HGD on 4T1 tumor-bearing mice. (A) Representative ex vivo fluorescent images and (B) quantification of the organs and tumors collected from the mice at 24 ​h post-injection of Cy5.5-labeled micelles. (C) Fluorescent images of tumor tissues from 4T1 orthotopic tumor-bearing mice after intravenous injection with DOX, ML-HGD, and PL-HGD (at 8 ​mg/kg DOX equivalent) for 24 ​h. The scale bar is 20 ​μm. Blue fluorescence represents the cell nucleus stained with DAPI. Red fluorescence represents the DOX released from micelles.

The tumor-bearing mice were intravenously injected with Cy5.5-labeled ML-HGD and PL-HGD to observe the distribution of the micelles in the organs and tumors. The tumor accumulation of PL-HGD was two times higher than ML-HGD at 24 ​h post-injection (Fig. 7A and B). In addition, DOX intensity from free DOX or micelles in the tumors were also evaluated after tumor-bearing mice were injected testing samples at a dose of 8 ​mg/kg equivalent of DOX for 24 ​h. The experimental results show that PL-HGD had a higher DOX signal compared to those of free DOX and ML-HGD (Fig. 7C and Fig. S15-19) because the exposed targeting ligands assisted the PL-HGD to accumulate in the tumors by preventing it from washing out of the tissues at the TME [51]. On the other hand, our previous study was showed that free DOX could accumulate in the liver and spleen at 24 ​h while the fluorescent intensity of DOX for those organs was low. At 72 ​h post-injection, an obvious fluorescent signal was observed in the liver and other organs [52]. Therefore, free DOX had a higher accumulation in the tumor than in other tissues at 24 ​h. Additionally, the hemocompatibility of micelles was assessed. Both ML-HGD and PL-HGD exhibited lower hemolysis than free DOX, indicating that our nanoparticles had high hemocompatibility (Fig. S20 and S21). PL-HGD had better stability, pH-responsiveness, cancer cell cytotoxicity, cytokine secretion, and tumor accumulation than ML-HGD. In addition, the concentration of encapsulated IMQ and conjugated DOX was difficult to adjusted to the same level for PL-HGD and ML-HGD. Therefore, PL-HGD was selected for further evaluation of antitumor activity. h.

**Fig. 8:**
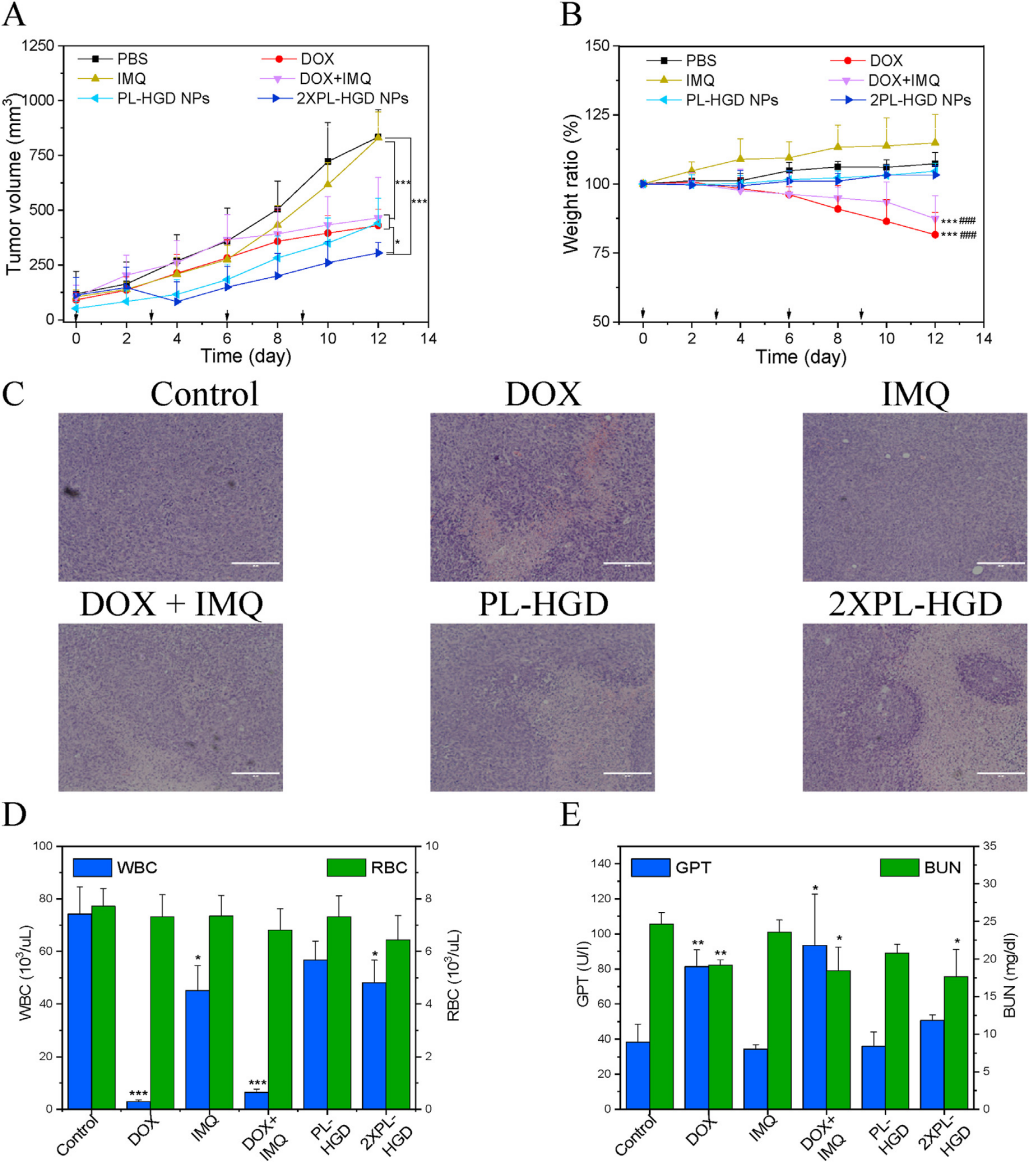
Therapeutic efficacy of PL-HGD. (A) The tumor volume and (B) body weight ratio of tumor-bearing mice after intravenous injection of drugs and micelles. The arrows mean drugs and micelles (at 8 ​mg/kg DOX equivalent) were administered on days 0, 3, 6, and 9. (C) Slice of tumor tissue stained with hematoxylin and eosin (H&E) after treatments. The scale bar is 200 ​μm. (D) The concentrations of white blood cells (WBCs)/concentration of red blood cells (RBCs) and (E) glutamic pyruvic transaminase (GPT)/blood urea nitrogen (BUN) for tumor-bearing mice after treatments. All data have been expressed as mean ​± ​SD from at least three independent experiments. (∗P ​< ​0.05, ∗∗P ​< ​0.01 and ∗∗∗P ​< ​0.001 as compared with control group; #P ​< ​0.05, ##P ​< ​0.01 and ###P ​< ​0.001 as compared with PL-HGD group).

For the antitumor study, the 4T1 orthotopic tumor-bearing mice were randomly divided to 6 groups and injected intravenously with PBS, DOX, IMQ, DOX combined with IMQ, PL-HGD, and 2-fold PL-HGD on days 0, 3, 6, and 9. The results of tumor growth inhibition show that 2-fold PL-HGD significantly reduced tumor growth without weight loss (Fig. 8A and B). PL-HGD could also inhibit tumor growth in the initial 8 days as well as 2-fold PL-HGD. As time increased, there were no significant differences in the treatments of DOX, DOX combined with IMQ, and PL-HGD on day 12. However, mice treated with DOX or DOX plus IMQ showed severe body weight loss. The tumor volume after free IMQ treatment was not significantly different (*P* ​= ​0.918) from the control group because the dose of IMQ administered via intravenous injection was too low to inhibit tumor growth in the 4T1 tumor-bearing mice [6]. In order to confirm the antitumor activity, tumor tissues from each group were stained with hematoxylin and eosin (H&E). Although the cell number was observed to be reduced in all treated groups, PL-HGD and 2-fold PL-HGD had better performance than other treatments (Fig. 8C). In contrast, DOX and DOX combined with IMQ induced higher levels of serum GPT and highly reduced WBC counts compared to those in the control group (Fig. 8D and E). The serum BUN in free DOX, DOX ​+ ​IMQ and PL-HGD group were significant difference compared to control group. Actually, the level of GOT, BUN and CRE were still within the normal range (Fig. 8E and Fig. S22) [53,54]. The major organs after PL-HGD treatment did not exhibit pathological differences compared with the control group, indicating that PL-HGD was relatively safe (Fig. S23). Although PL-HGD exhibited similar antitumor activity with DOX and DOX combined with IMQ, weight loss and WBC reduction were not observed. These adverse effects are the major reasons for chemotherapy failure, limiting the use of the chemotherapeutic agents [55]. In this study, PL-HGD exhibited lower cytotoxic effects and similar therapeutic efficacy compared to those of DOX and DOX combined with IMQ. Although the antitumor efficacy of PL-HGD significantly improved upon increasing the dose up two-fold (2-fold PL-HGD), the mice did not exhibit any weight loss, high serum GPT levels, and WBC reduction.

**Fig. 9:**
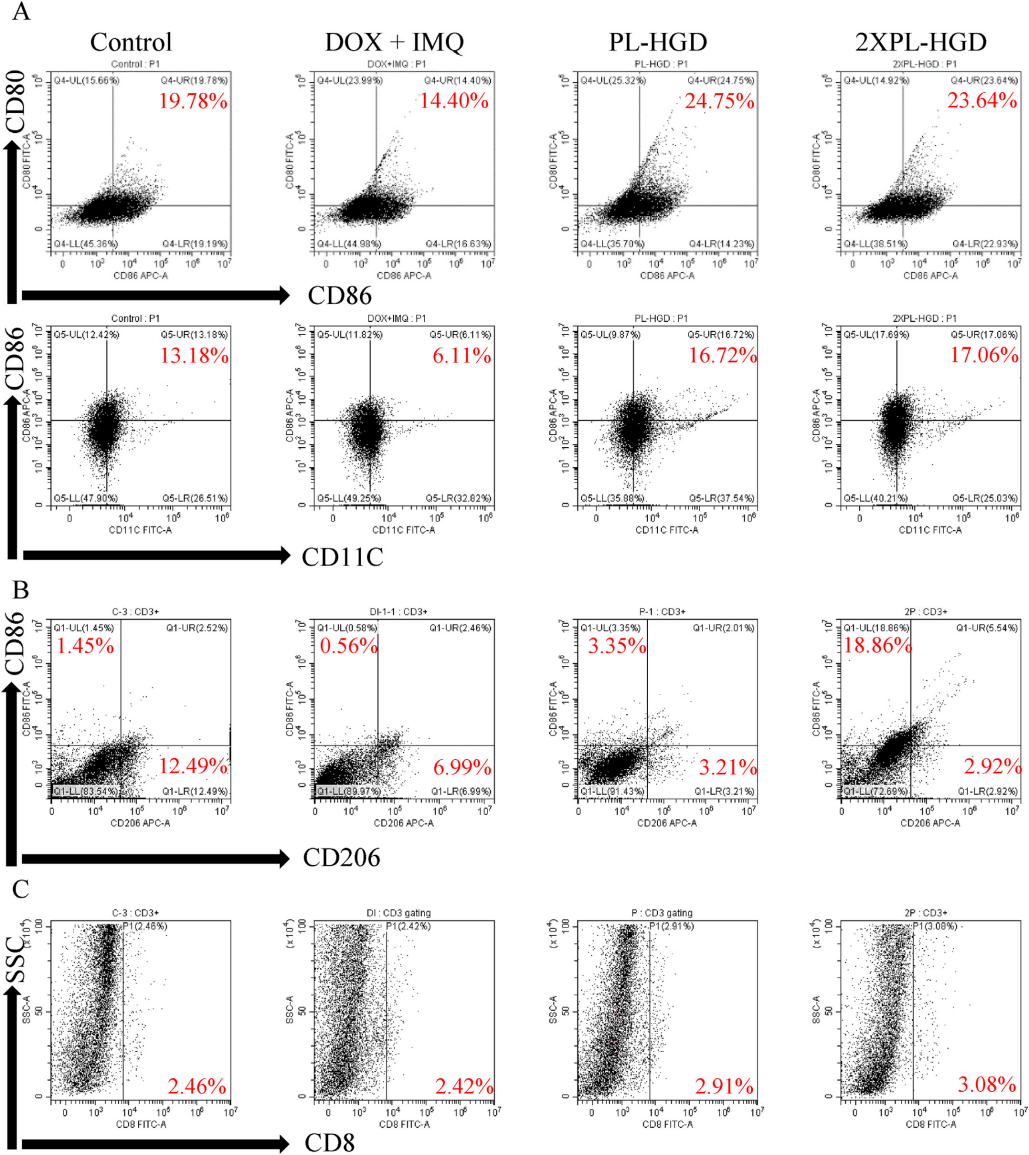
Immune statuses of tumor-bearing mice after treatments. (A) The expressions of CD80, CD86, and CD11C antibodies in immune cells obtained from right inguinal lymph nodes. (B) The population of CD86^+^ (M1) or CD206^+^ (M2) cells in F4/80 dual positive cells and (C) CD8^+^ cells gated on CD3^+^ T cells in tumor tissues after treatments for 12 days.

**Fig. 10:**
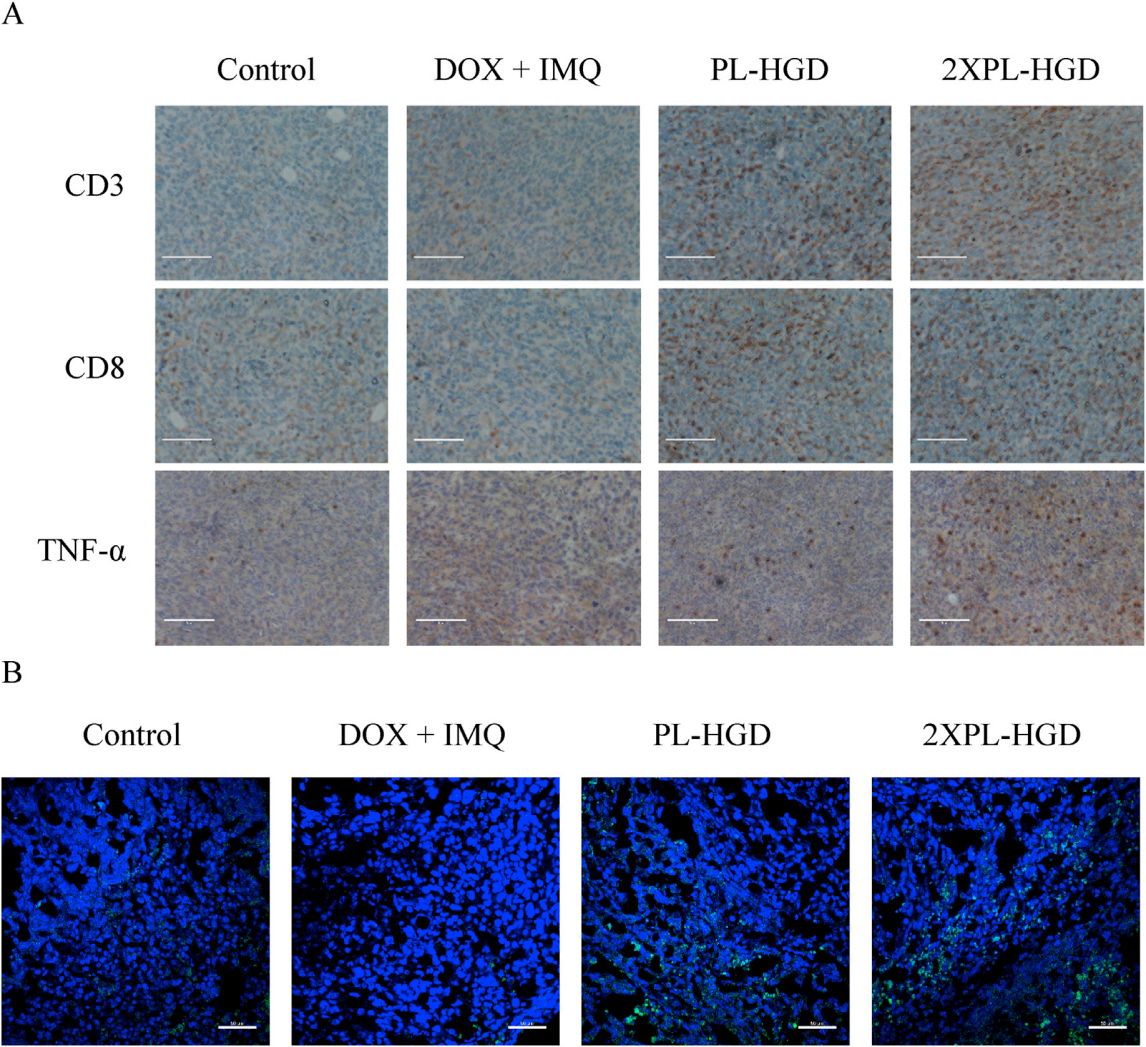
Immunostaining of tumor tissues in tumor-bearing mice after treatments. (A) Immunohistochemistry images of tumor sections stained with CD3, CD8, and TNF-α antibodies. The scale bar is 100 ​μm. (B) Immunofluorescence images of tumor tissues after treatments for 12 days. The scale bar is 50 ​μm. Blue fluorescence represents the cell nucleus stained with DAPI. Green fluorescence represents the iNOS stained with the iNOS antibody conjugated with FITC.

As the DCs sense cancer antigens mediated by IMQ, they can transform from their immature state to an activated form [6]. Activated DCs have been shown to express high levels of CD80, CD86, CD83, and CD40 on cellular membrane [56,57]. Reports have also shown that activated DCs can migrate to lymph nodes in order to present antigens to T cells through the T-cell receptors [58], In order to evaluate the immune status, mice were sacrificed after 12 days of treatments. The inguinal lymph nodes and tumors were collected, dissociated, and co-stained with specific marker. Furthermore, tumor tissue slices were also stained with anti-CD3 and anti-CD8, which are important biomarkers of cytotoxic T cells [59]. In this study, mice that received DOX combined with IMQ treatment exhibited lower CD80, CD86, and CD11C expressions compared to those of the control group. In contrast, PL-HGD and 2-fold PL-HGD were able to increase the percentages of CD80^+^, CD86^+^, and CD11C^+^ cells, indicating that they could facilitate the transformation of DCs into their mature form in lymph nodes (Fig. 9A). In addition, tumor tissue after 2-fold PL-HGD treatment had the highest M1/M2 ratio (around 6.46); the M1/M2 ratios for the control, free DOX ​+ ​IMQ, PL-HGD and 2-fold PL-HGD were 0.12, 0.08, 1.04 and 6.45, respectively (Fig. 9B). The experimental result indicated 2-fold PL-HGD not only dramatically increased the percentages of F4/80^+^/CD86^+^ cells (M1 macrophages) but decreased the population of F4/80^+^/CD206^+^ cells (M2 macrophages). The population of CD3^+^/CD8^+^ cells increased after PL-HGD treatment (Fig. 9C); the IMQ induced upregulation of the costimulatory signal (CD80/CD86) on the surface of the dendritic cell to stimulate CD8^+^ T cell activation [[60], [61], [62]]. On the other hand, the immunohistochemical staining of the tumor sections revealed that both CD3^+^ and CD8^+^ T cells dramatically increased after treatments with PL-HGD and 2-fold PL-HGD (Fig. 10A), demonstrating that activated cytotoxic T cells recognized specific antigens presented by activated DCs and penetrated into the tumors. Upon treatment with PL-HGD and 2-fold PL-HGD, the expression of TNF-α and iNOS were notably increased (Fig. 10A,B and Fig. S24). Immune cells within the TME are crucial for an anti-cancer effect. Previous studies have shown that high densities of CD3^+^ and CD8^+^ T cells in the tumor interior and the invasive margin region indicate lower tumor recurrence [63]. Proinflammatory cytokines including TNF-α and iNOS have been shown as important markers of M1-like macrophages [64,65]. Polarization of macrophages from M2 to M1-type has been shown to contribute to antitumor immunity and inhibition of angiogenesis [66]. In addition, high M1/M2 ratio in tumor tissue exhibits better patients' survival time and prognosis [67]. Our antitumor efficacy results reveal that PL-HGD could deliver required dosages without causing adverse effects, inhibit tumor growth, and trigger immune responses for combined immunotherapy, which did not occur with DOX and IMQ treatments.

## 4 Conclusion

In this study, dual pH-sensitive and TME-active targeting micelles were designed for combined chemo- and immunotherapy to treat cancer. These micelles could exactly expose glucose ligands and positive charges to target cancer cells in the TME and release DOX into cancer cells for chemotherapy. Furthermore, these micelles also released IMQ inside TAMs and in the TME, thereby polarizing TAMs towards the M1-like phenotype for macrophage-mediated immunotherapy and maturing DCs for immunotherapy. Our results demonstrated that dual pH-sensitive and TME-active targeting micelles were selectively toxic to 4T1 cells without damaging normal cells and macrophages and exhibited high tumor accumulation, resulting in significant tumor growth inhibition and fewer adverse effects. Micelles could also induce high percentages of mature DCs in the lymph node and increase the densities of CD3^+^, CD8^+^ T cells, as well as M1-like macrophages in tumor tissues. Although treatment with these dual pH-sensitive and TME-active targeting micelles did not completely destroy all cancer cells, it demonstrated that precisely designing the structure of nanomedicines can achieve a therapeutic effect, validating this multi-treatment approach to treat cancer.

## Credit author statement

**Yu-Han Wen:** Conceptualization, Methodology, Investigation, Visualization, Writing - Original Draft, Writing - Review & Editing. **Po–I Hsieh:** Investigation, Visualization. **Hsin-Cheng Chiu:** Conceptualization, Supervision. **Chil-Wei Chiang:** Investigation. **Chun-Liang Lo:** Conceptualization, Visualization, Supervision, Project administration, Writing - Review & Editing. **Yi-Ting Chiang:** Conceptualization, Supervision, Resources.

## Declaration of competing interest

The authors declare that they have no known competing financial interests or personal relationships that could have appeared to influence the work reported in this paper.

## Data Availability

Data will be made available on request.
