# Supplementary material for: Precise delivery of doxorubicin and imiquimod through pH-responsive tumor microenvironment-active targeting micelles for chemo- and immunotherapy
Source: Mater Today Bio. 2022 Nov 3;17:100482. doi: 10.1016/j.mtbio.2022.100482 (PMC9647582; doi:10.1016/j.mtbio.2022.100482)
Supplement: Multimedia component 1 [file mmc1.docx]

Supporting Information

**Precise Delivery of Doxorubicin and Imiquimod through pH-Responsive Tumor Microenvironment-Active Targeting Micelles for Chemo- and Immunotherapy**

*Yu-Han Wen^a,b^, Po-I Hsieh^a,b^, Hsin-Cheng Chiu^c^, Chil-Wei Chiang^a,b^, Chun-Liang Lo^a,b,*^, Yi-Ting Chiang^d*^*

^a^ Department of Biomedical Engineering, National Yang Ming Chiao Tung University, Taipei 112, Taiwan, ROC

^b^ Medical Device Innovation and Translation Center, National Yang Ming Chiao Tung University, Taipei 112, Taiwan, ROC

^c^ Department of Biomedical Engineering and Environmental Sciences, National Tsing-Hua University, Hsinchu 300, Taiwan, ROC

^d^ School of Pharmacy, China Medical University, Taichung 406040, Taiwan, ROC

Corresponding author.

E-mail: [cllo@nycu.edu.tw](mailto:cllo@nycu.edu.tw), [ytchiang@mail.cmu.edu.tw](mailto:ytchiang@mail.cmu.edu.tw)

**Table S1.** The composition and characterization of P(MAA-*co*-NHS)MPA and P(PAA-*co*-NHS)MPA

| Code | In feed  (mol %) | | |  | In copolymer  (mol %)^a^ | | | Mw^b^ | PDI^b^ |
| --- | --- | --- | --- | --- | --- | --- | --- | --- | --- |
|  | MAA | PAA | NHS |  | MAA | PAA | NHS |  |  |
| P(MAA-*co*-NHS) MPA | 27.5 | --- | 72.5 |  | 37.0 | --- | 63.0 | 13300 | 6.49 |
| P(PAA-*co*- NHS) MPA | --- | 65 | 35 |  | --- | 21.9 | 78.1 | 12400 | 2.37 |

^a^ The molar percentages of the methacrylic acid (MAA), propylacrylic acid (PAA) and MAA-NHS (NHS) were calculated from ^1^H-NMR spectrum.

^b^ The average molecular weight (Mw) and polydispersity index (PDI) of copolymers were measured by GPC using PEG as a standard.

**Table S2.** The composition and characterization of PM-HGD and PP-HGD

| Code | In feed  (mol %) | | |  | In copolymer  (mol %) | | | | Mw^b^ | PDI^b^ |
| --- | --- | --- | --- | --- | --- | --- | --- | --- | --- | --- |
|  | HIS | GLU | DOX |  | HIS^a^ | GLU^a^ | DOX^a^ | DOX ^c^ |  |  |
| M-HGD | 25.2 | 12.6 | 25.2 |  | 18 | 16 | 19 | 19.1 | 1270 | 1.44 |
| P-HGD | 31.2 | 15.6 | 31.2 |  | 35 | 32 | 23 | 20.7 | 1430 | 1.43 |

^a^ The molar percentages of the histidine (HIS), glucosamine (GLU) and doxorubicin (DOX) were calculated from ^1^H-NMR spectrum.

^b^ The Mw and PDI of copolymers were measured by GPC using PEG as a standard.

^c^ The DOX concentration was quantified by UV-Vis spectroscopy using free DOX as a standard.

**Table S3.** Characteristics of M-HGD and P-HGD core nanoparticles

|  | In feed | | |  | Particle characteristics^a^ | | |
| --- | --- | --- | --- | --- | --- | --- | --- |
| Code | M-HGD copolymer  (mg) | P-HGD copolymer  (mg) | IMQ  (mg) |  | Size  (nm) | PDI | Zeta potential (mv) |
| M-HGD | 15 | --- | 2 |  | 159.4  ± 1.7 | 0.456  ± 0.029 | -31.1  ± 1.5 |
| P-HGD | --- | 15 | 2 |  | 134.6  ± 11.5 | 0.202  ± 0.025 | -28.3  ± 4.2 |

^a^ Characteristics of core nanoparticles were determined by DLS.


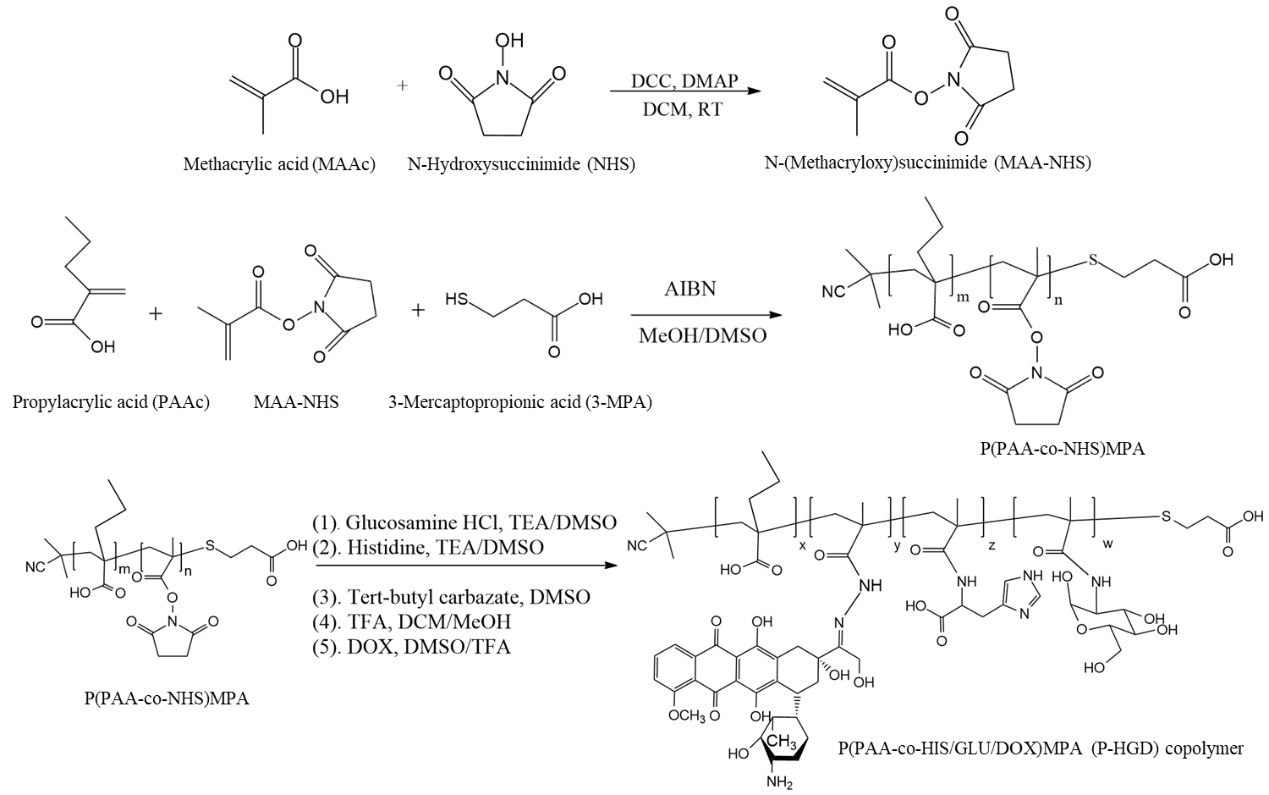


**Fig. S1.** Synthesis scheme of P(PAA-*co*-HIS/GLU/DOX)MPA (P-HGD) copolymers.

| A | B |
| --- | --- |
| 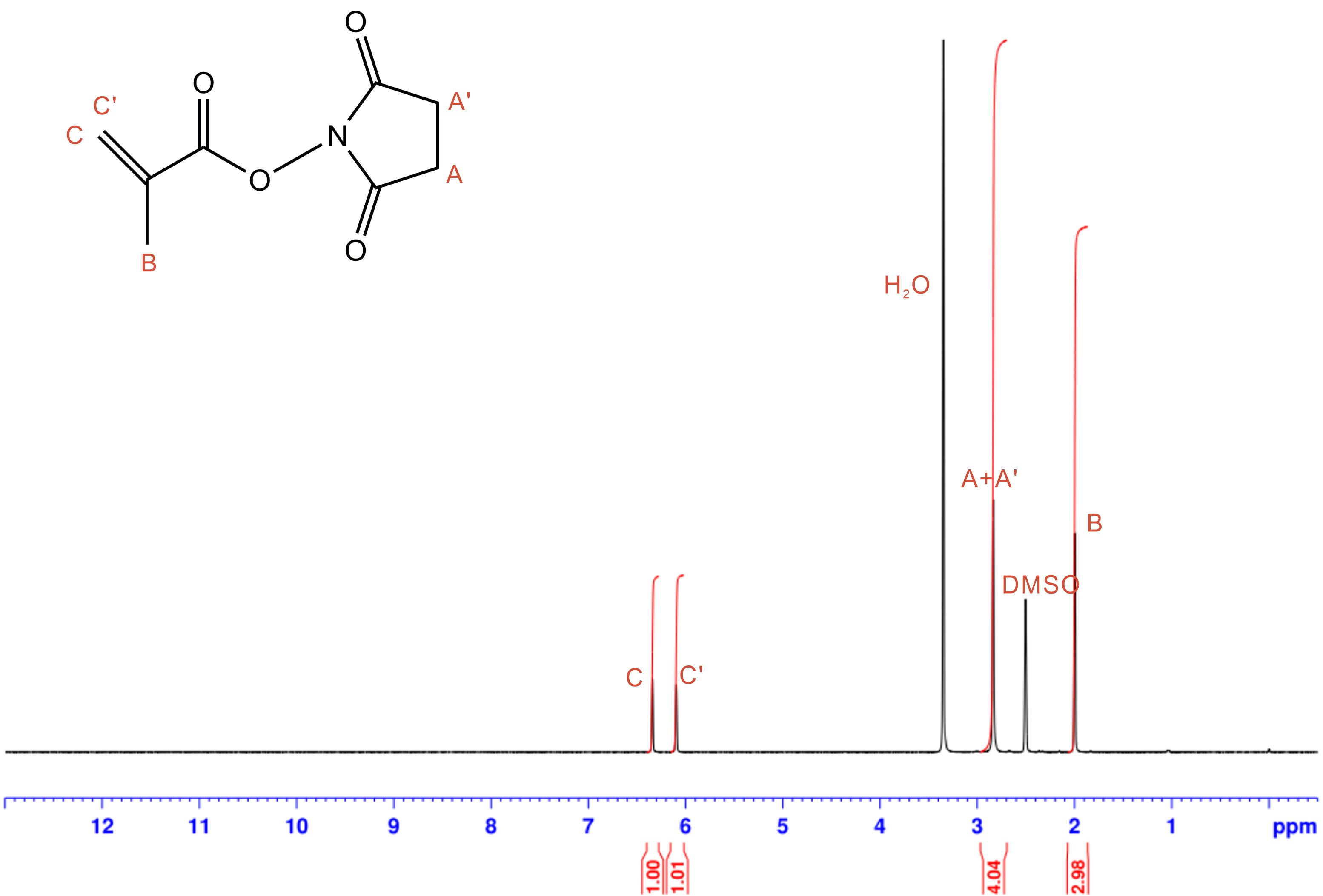 |  |

**Fig. S2.** (A) ^1^H-NMR and (B) FT-IR spectrum of MAA-NHS.

| A | B |
| --- | --- |
| 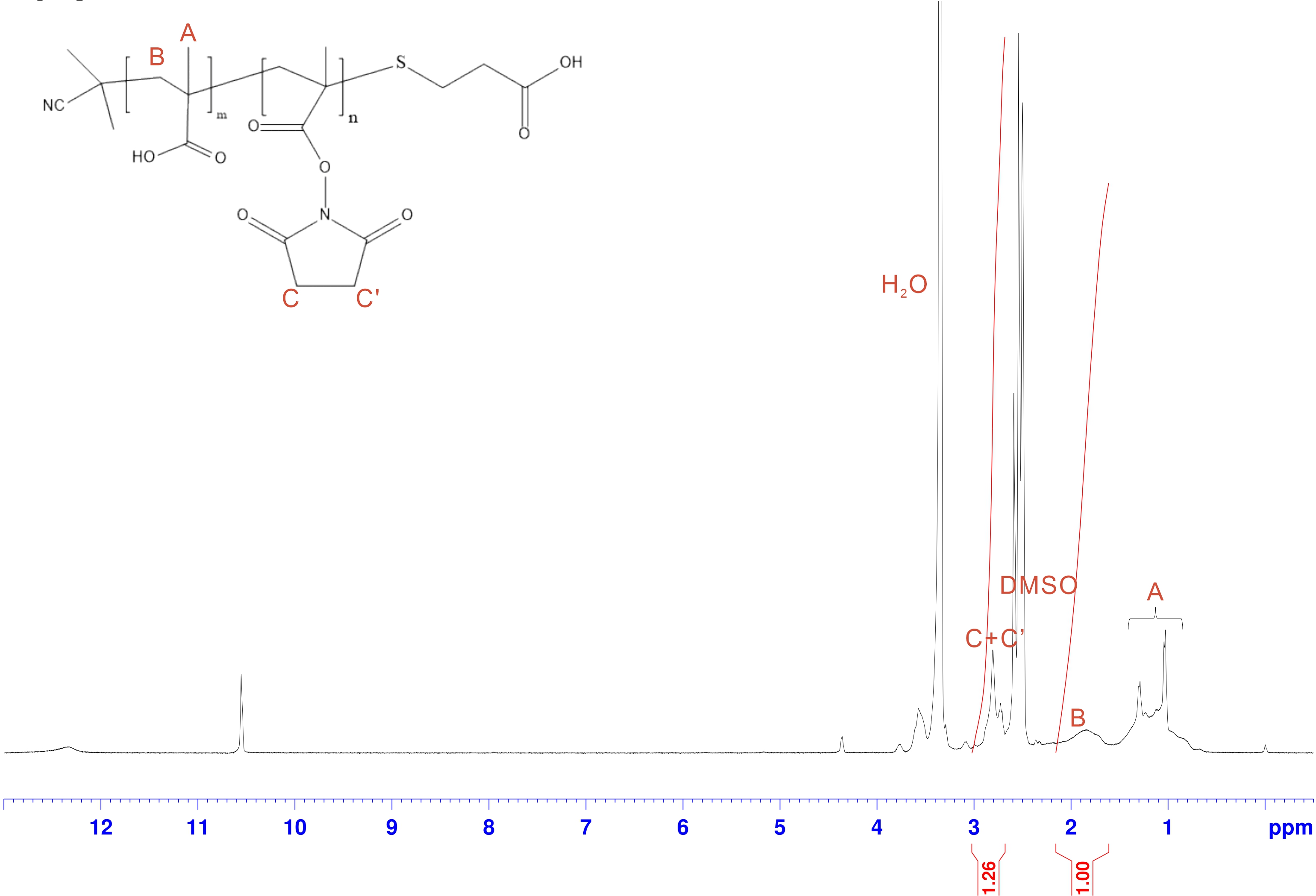 |  |

**Fig. S3.** (A) ^1^H-NMR and (B) FT-IR spectrum of P(MAA-*co*-NHS)MPA.

| A | B |
| --- | --- |
| 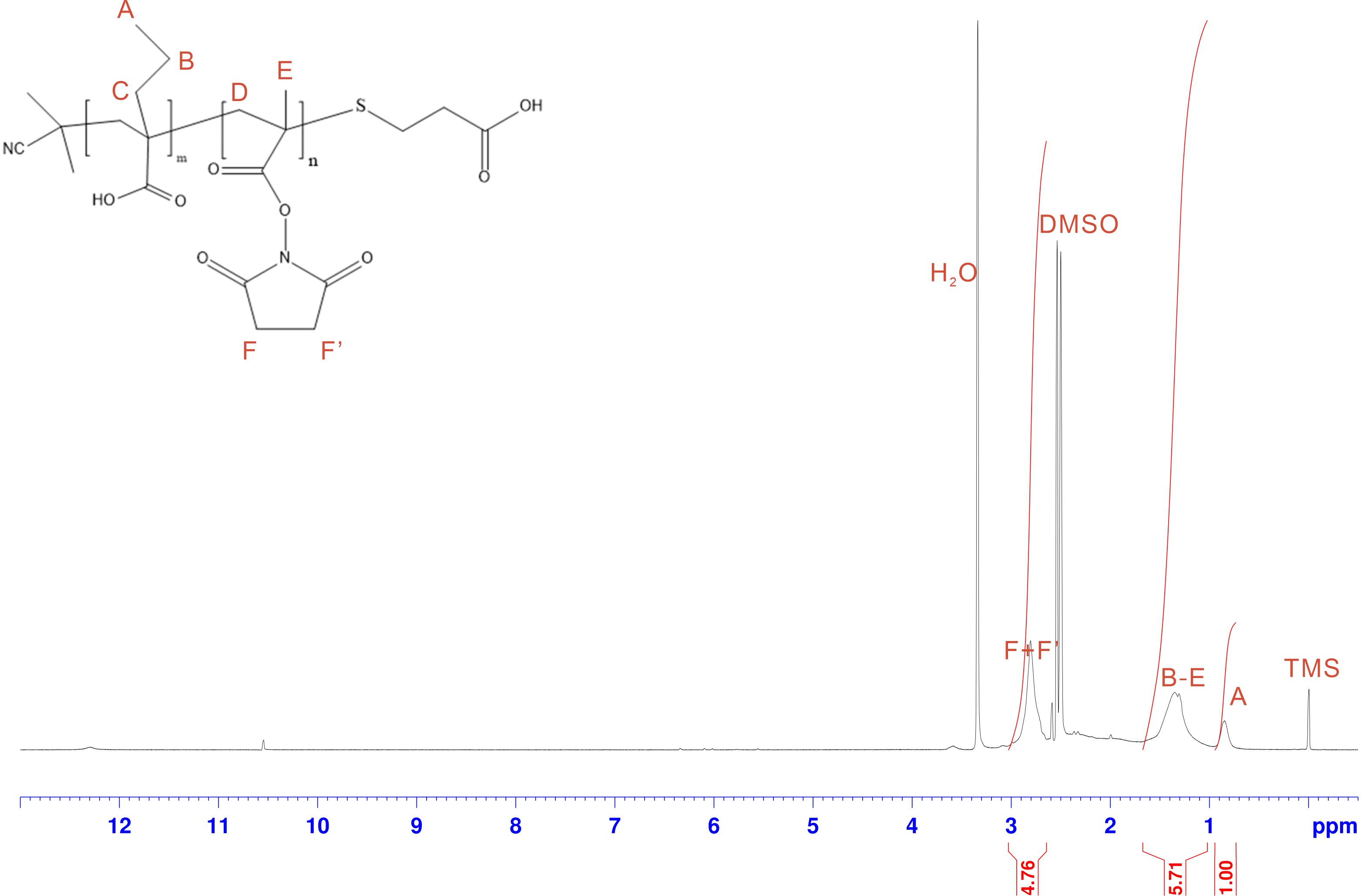 |  |
|  | |

**Fig. S4.** (A) ^1^H-NMR and (B) FT-IR spectrum of P(PAA-*co*-NHS)MPA.

| A | B |
| --- | --- |
| 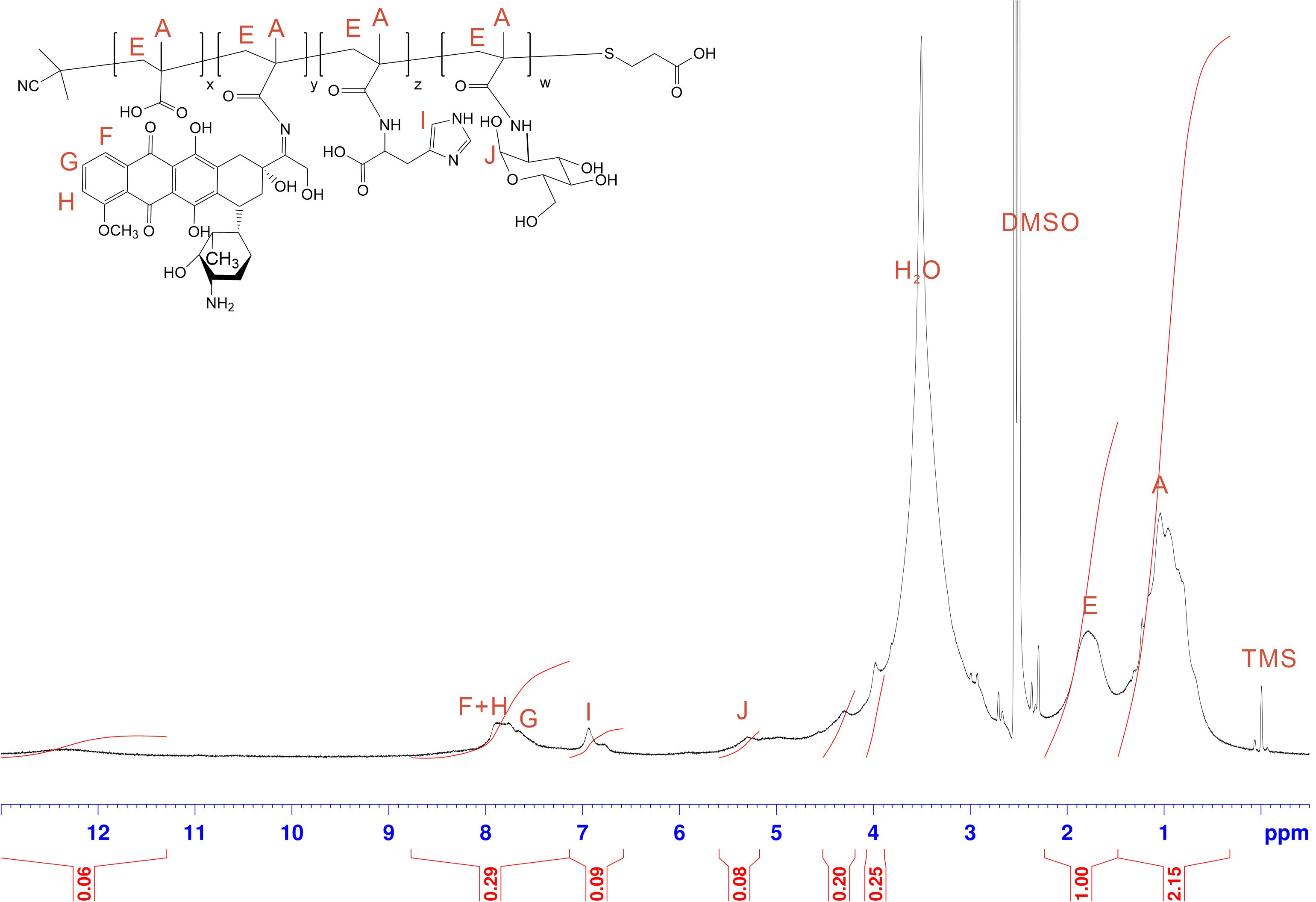 |  |
| **Fig. S5.** (A) ^1^H-NMR and (B) FT-IR spectrum of M-HGD. | |

| A | B |
| --- | --- |
| 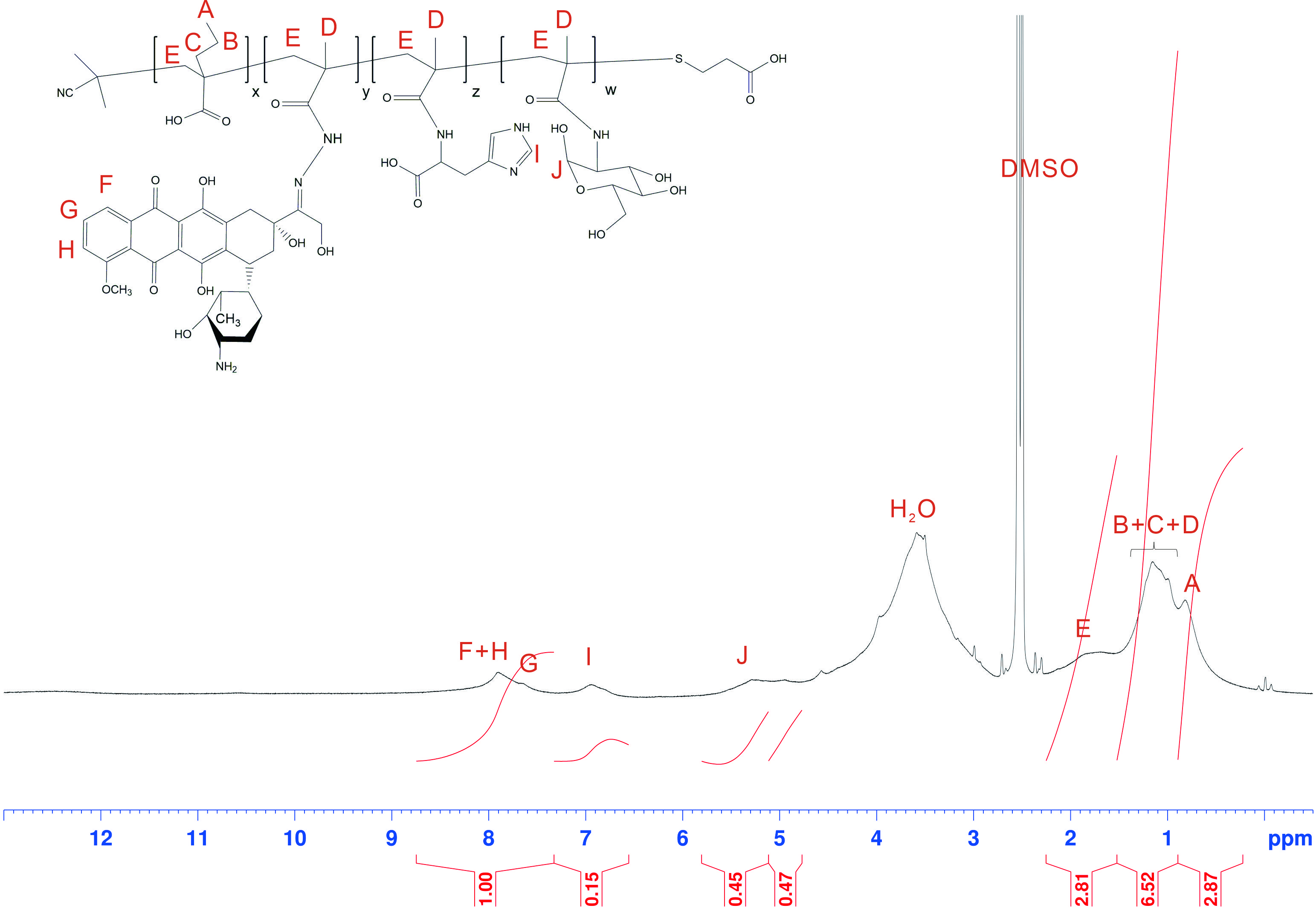 |  |
| **Fig. S6.** (A) ^1^H-NMR and (B) FT-IR spectrum of P-HGD. | |

| A | B |
| --- | --- |
|  |  |

**Fig. S7.** The GPC spectrum of (A) P(MAA-*co*-NHS)MPA/P(PAA-*co*-NHS)MPA and

(B) M-HGD/P-HGD copolymers.

**Fig. S8.** Titration curve of M-HGD and P-HGD copolymers

| A | B |
| --- | --- |
| 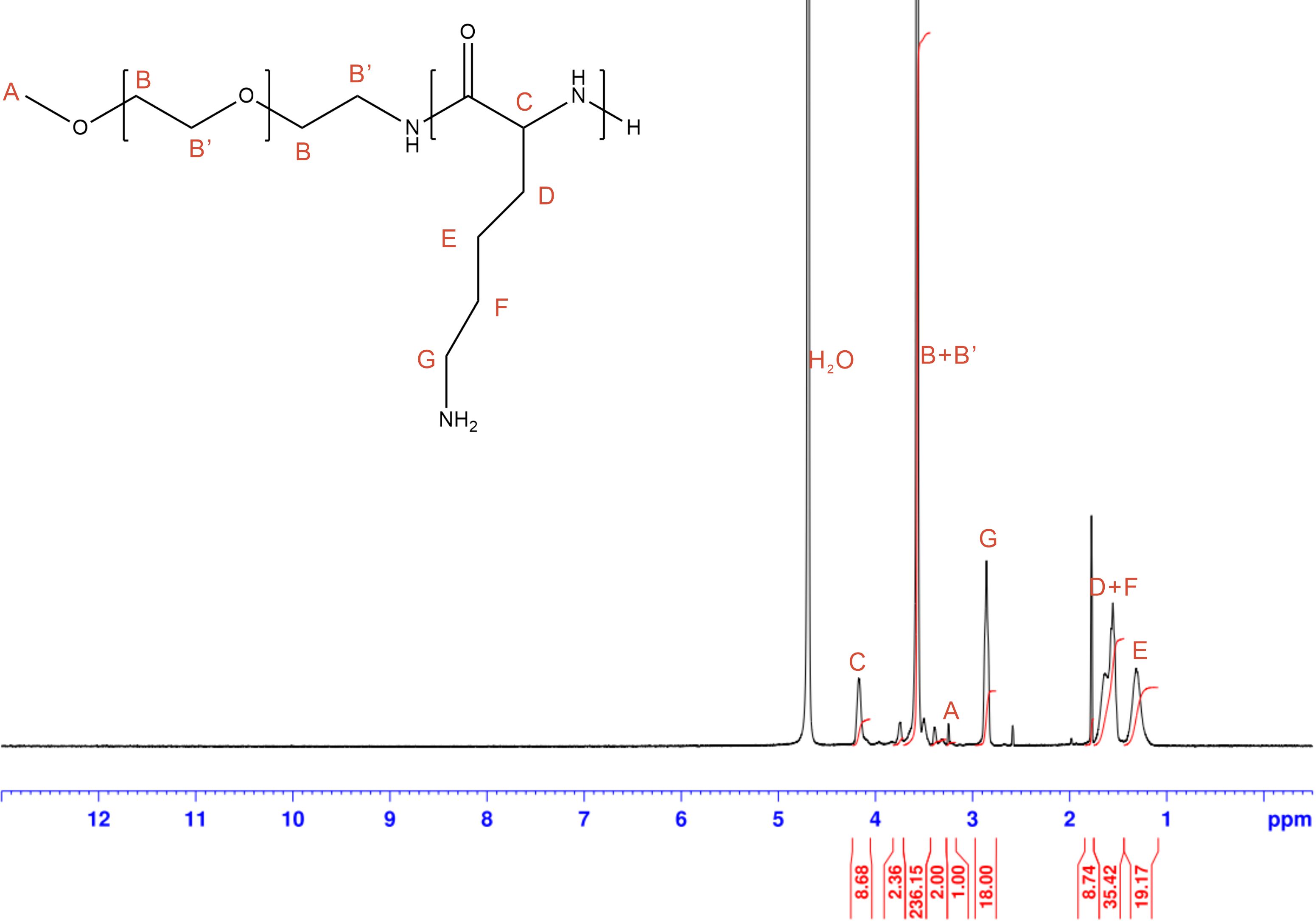 |  |

**Fig. S9.** (A) ^1^H-NMR and (B) FT-IR spectrum of mPEG-*b*-PLys.

| A | B |
| --- | --- |
|  |  |

**Fig. S10.** The TEM morphology of (A) ML-HGD and (B) PL-HGD micelles.

| A | B |
| --- | --- |
|  |  |
|  |  |
|  |  |

**Fig. S11.** Intensity distributions of ML-HGD (A) and PL-HGD (B) micelles from different batch.

| A | B |
| --- | --- |
|  |  |

**Fig. S12.** (A) UV-Vis absorption and (B) fluorescence emission spectra of free DOX, ML-HGD and PL-HGD

|  | ML-HGD | PL-HGD |
| --- | --- | --- |
| pH7.4 | 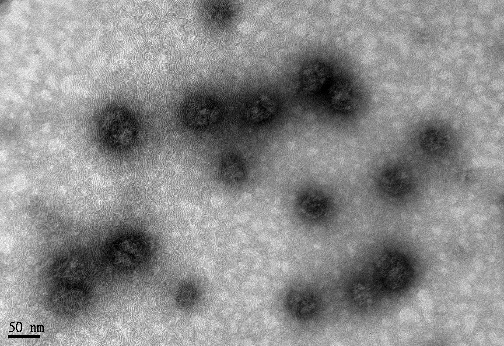 | 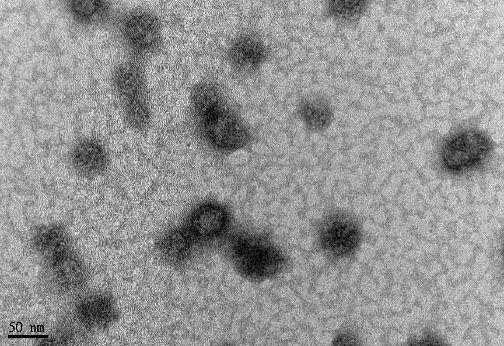 |
| pH6.5 | 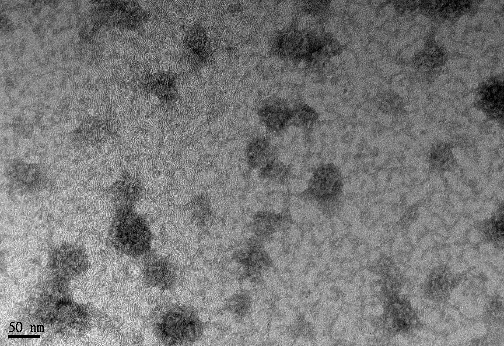 | 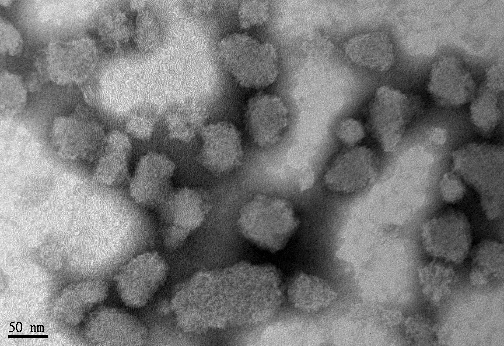 |
| pH6.0 | 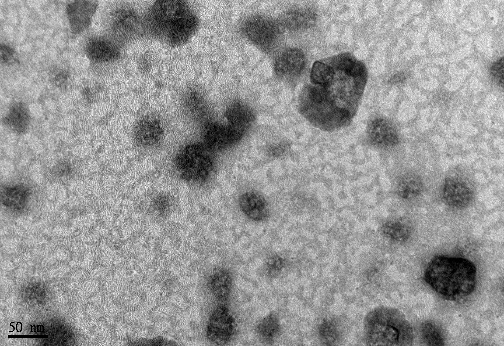 | 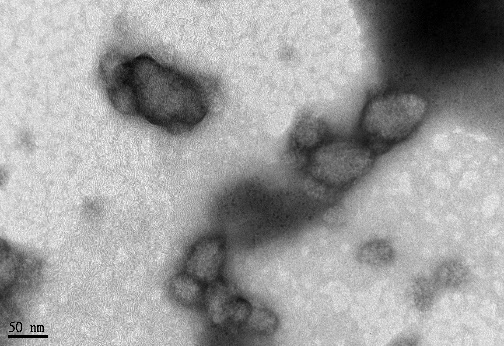 |
| pH5.0 | 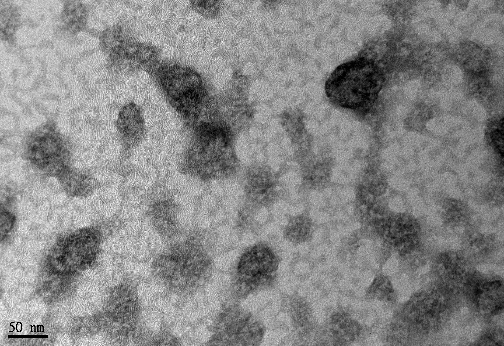 | 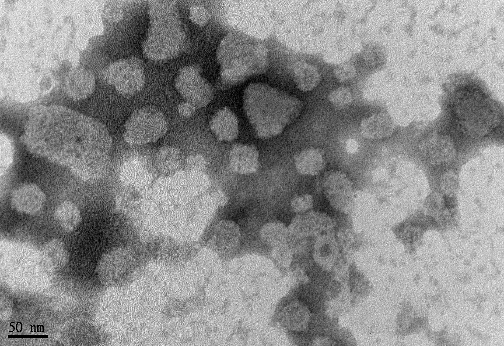 |

**Fig. S13.** TME image of ML-HGD and PL-HGD at various pH surrounding after 24 h of incubation. The scale bar is 50 nm.

A

B


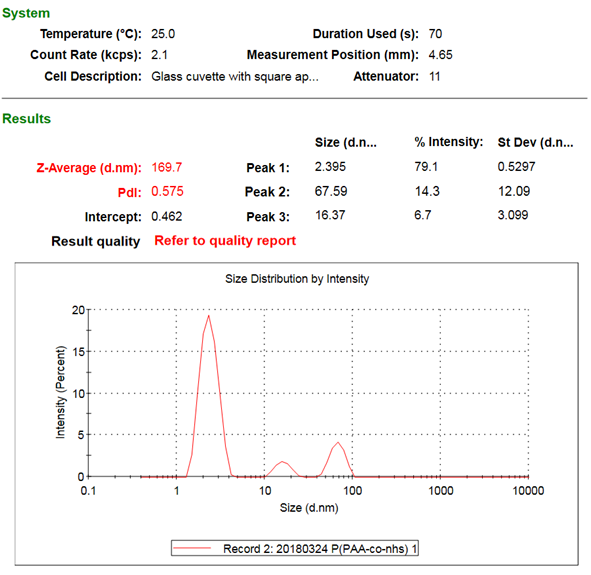

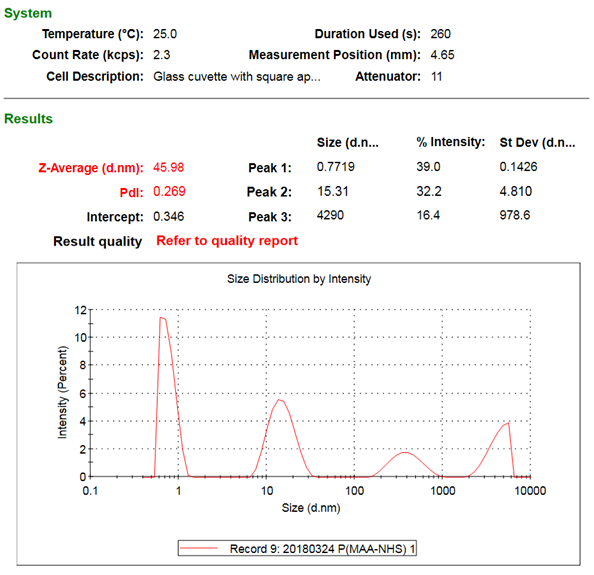


D

C


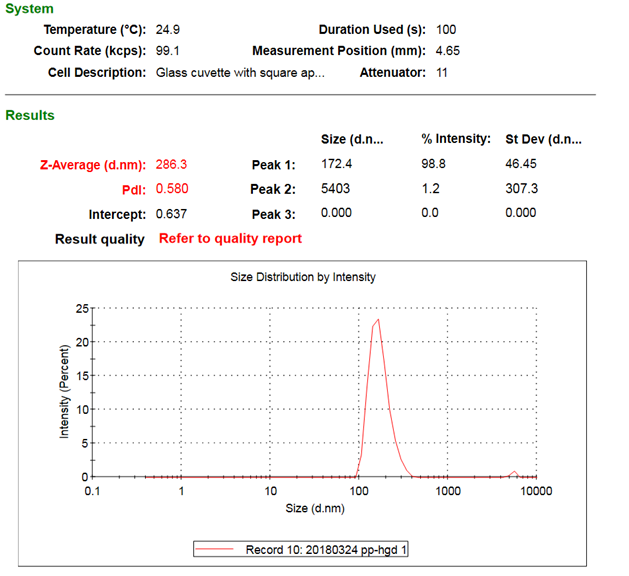

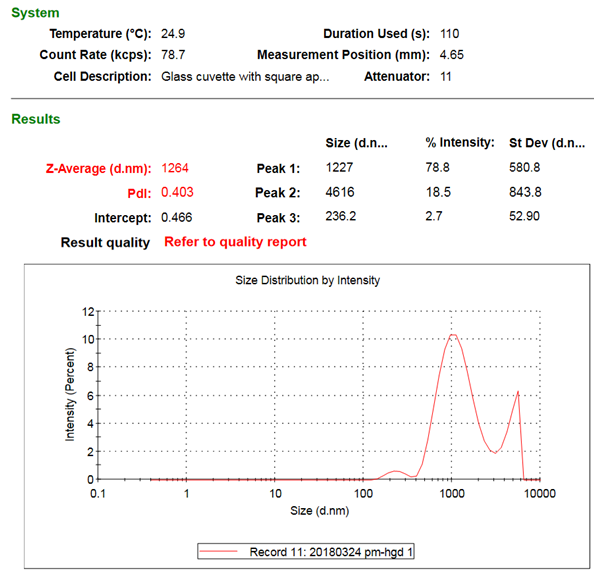


**Fig. S14.** The size distribution of (A) P(MAA-*co*-NHS)MPA, (B) P(PAA-*co* NHS)MPA, (C) M-HGD, and (D) P-HGD copolymers by DLS determination.

|  | DAPI | DOX | Merge |
| --- | --- | --- | --- |
| Free DOX | 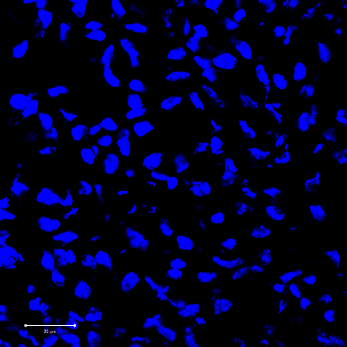 | 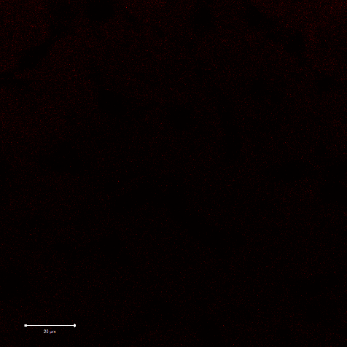 | 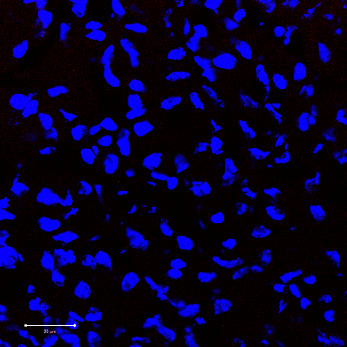 |
| ML-HGD | 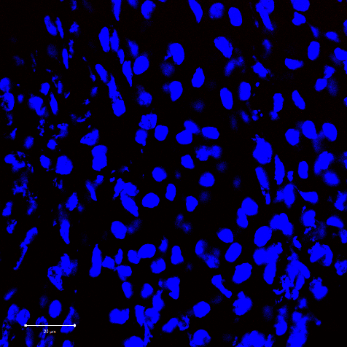 | 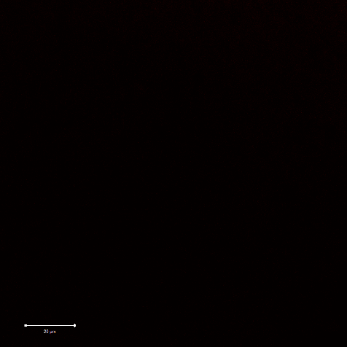 | 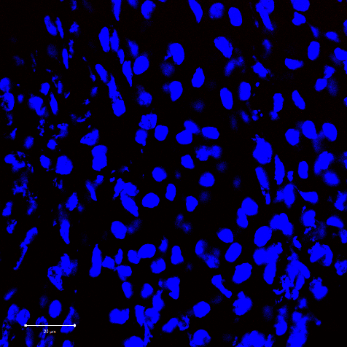 |
| PL-HGD | 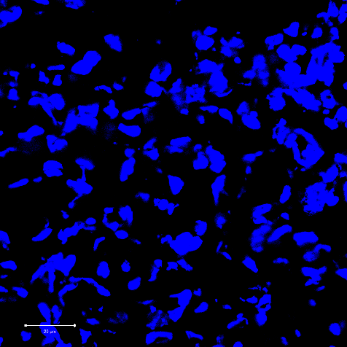 | 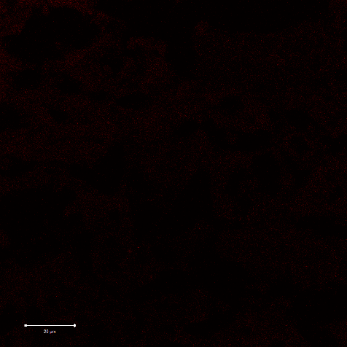 | 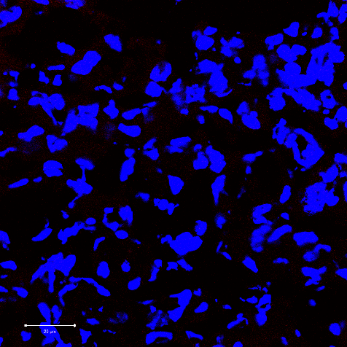 |

**Fig. S15.** Confocal images of kidney after intravenous injection of free DOX and micelles (at 8 mg/kg DOX equivalent) in 4T1 orthotopic tumor-bearing balb/c mice model for 24 h. The scale bar is 20 μm.

|  | DAPI | DOX | Merge |
| --- | --- | --- | --- |
| Free DOX | 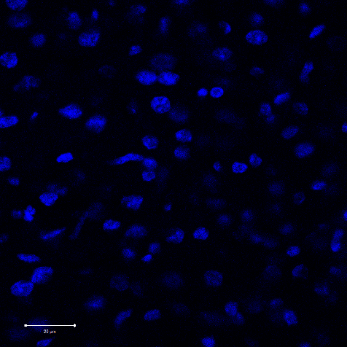 | 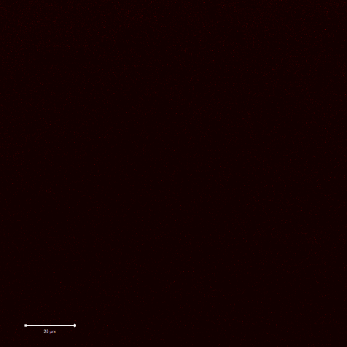 | 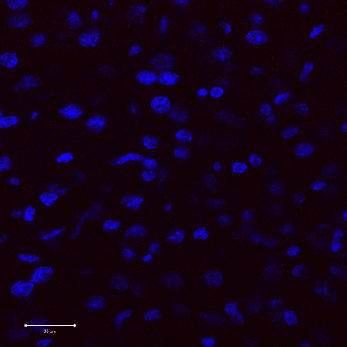 |
| ML-HGD | 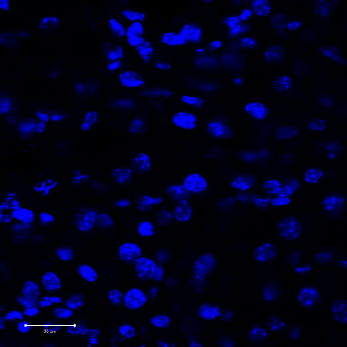 | 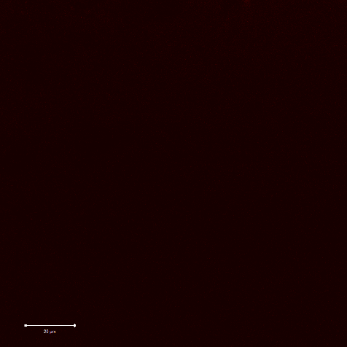 | 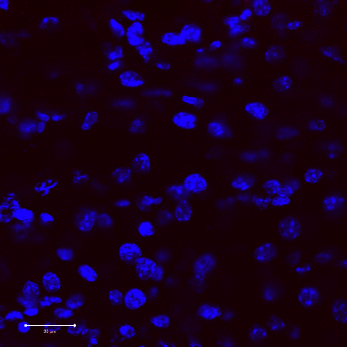 |
| PL-HGD | 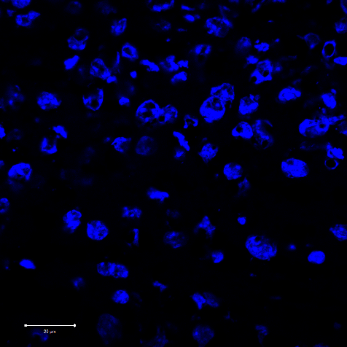 | 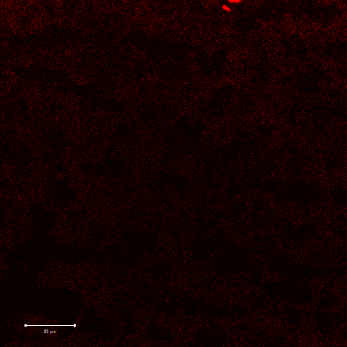 | 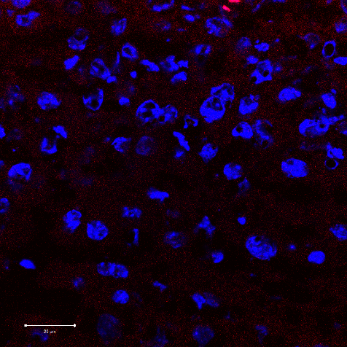 |

**Fig. S16.** Confocal images of liver after intravenous injection of free DOX and micelles (at 8 mg/kg DOX equivalent) in 4T1 orthotopic tumor-bearing balb/c mice model for 24 h. The scale bar is 20 μm.

|  | DAPI | DOX | Merge |
| --- | --- | --- | --- |
| Free DOX | 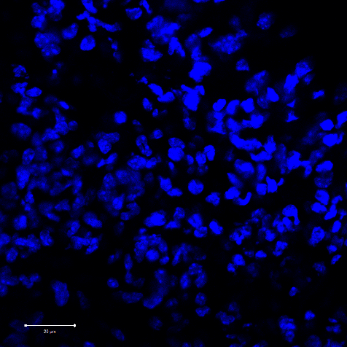 | 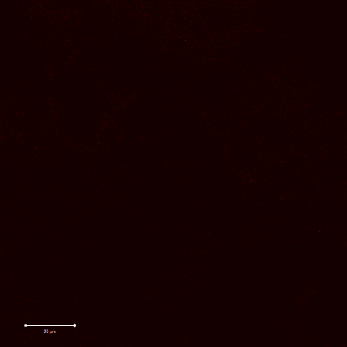 | 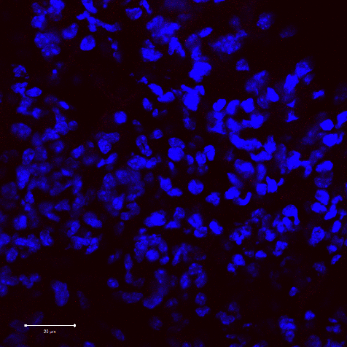 |
| ML-HGD | 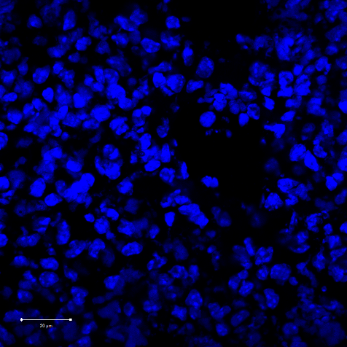 | 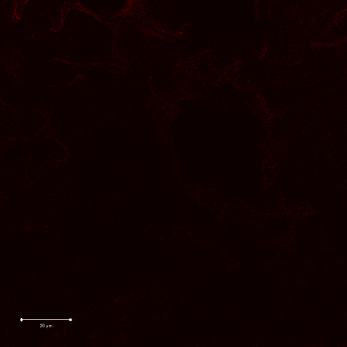 | 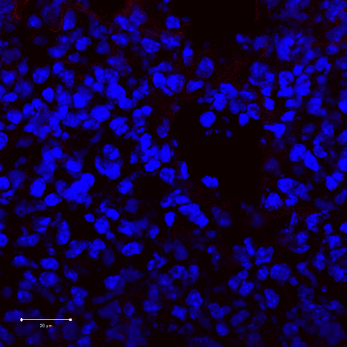 |
| PL-HGD | 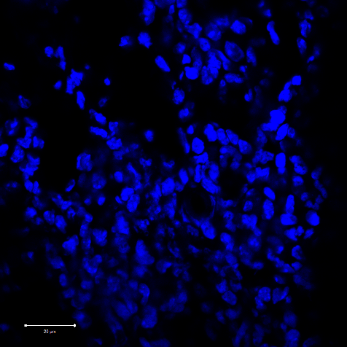 | 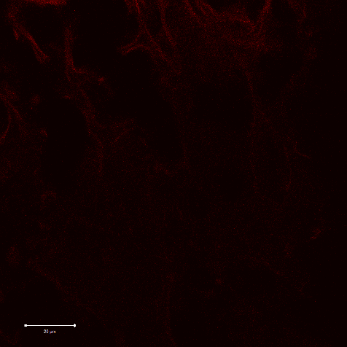 | 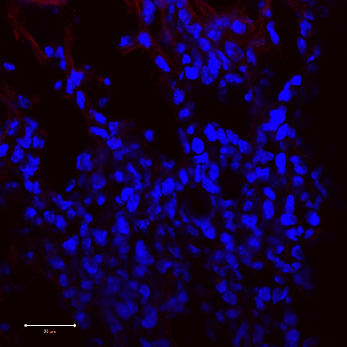 |

**Fig. S17.** Confocal images of lung after intravenous injection of free DOX and micelles (at 8 mg/kg DOX equivalent) in 4T1 orthotopic tumor-bearing balb/c mice model for 24 h. The scale bar is 20 μm.

|  | DAPI | DOX | Merge |
| --- | --- | --- | --- |
| Free DOX | 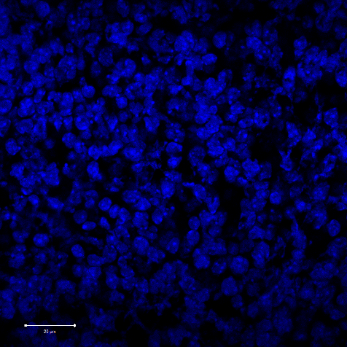 | 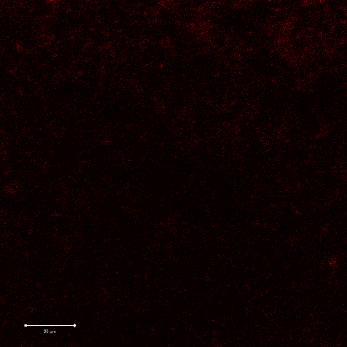 | 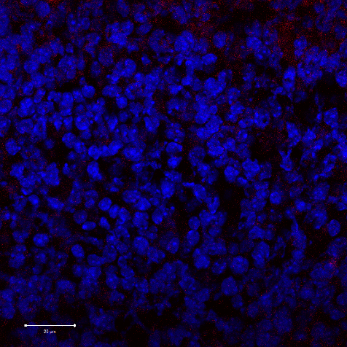 |
| ML-HGD | 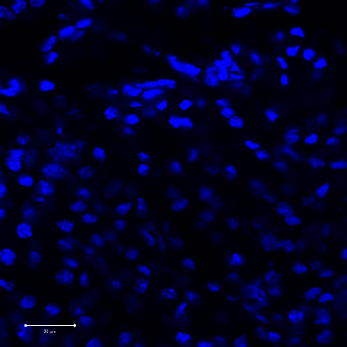 | 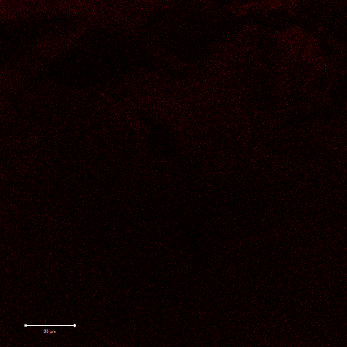 | 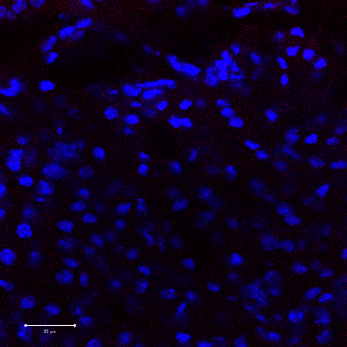 |
| PL-HGD | 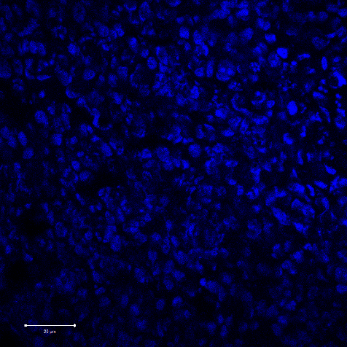 | 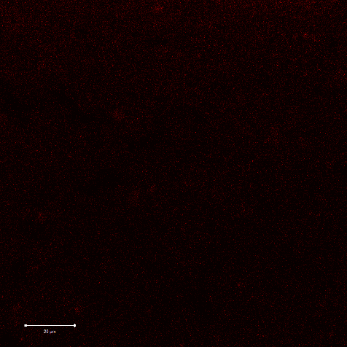 | 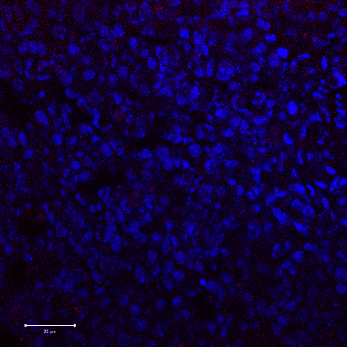 |

**Fig. S18.** Confocal images of spleen after intravenous injection of free DOX and micelles (at 8 mg/kg DOX equivalent) in 4T1 orthotopic tumor-bearing balb/c mice model for 24 h. The scale bar is 20 μm.

|  | DAPI | DOX | Merge |
| --- | --- | --- | --- |
| Free DOX | 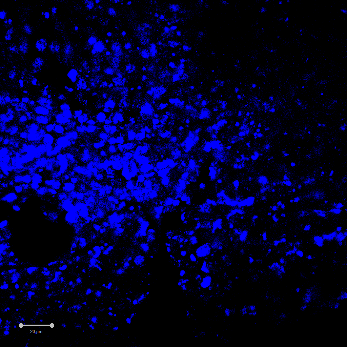 | 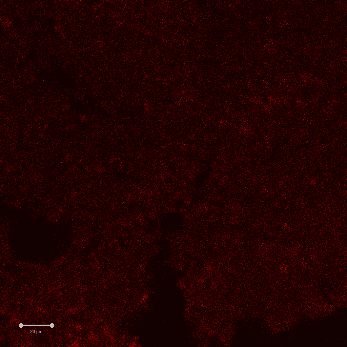 | 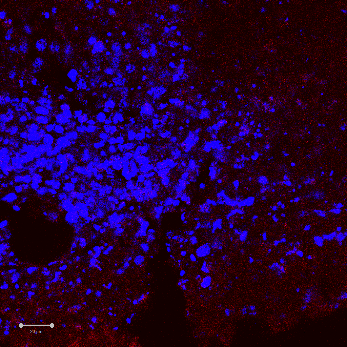 |
| ML-HGD | 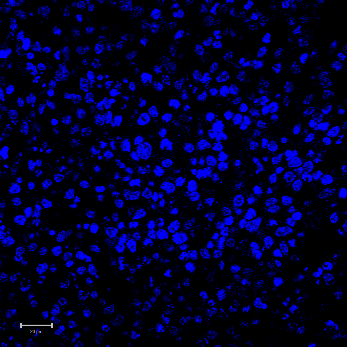 | 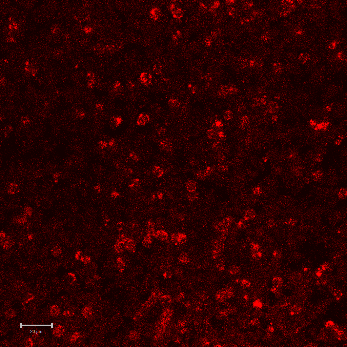 | 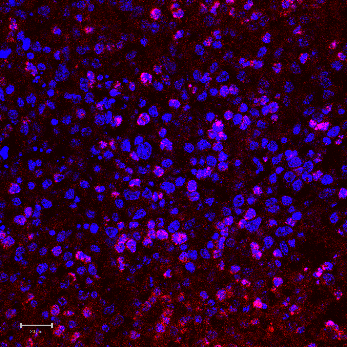 |
| PL-HGD | 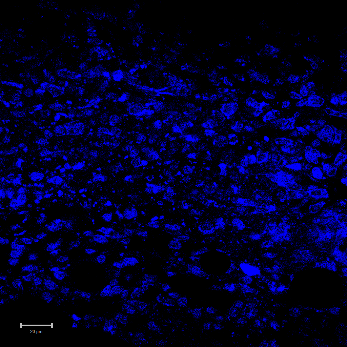 | 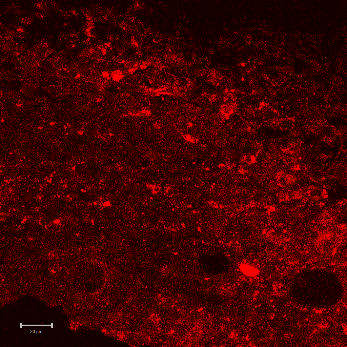 | 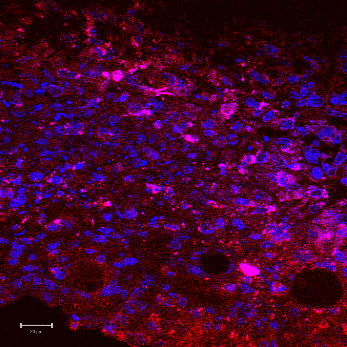 |

**Fig. S19.** Confocal images of tumor after intravenous injection of free DOX and micelles (at 8 mg/kg DOX equivalent) in 4T1 orthotopic tumor-bearing balb/c mice model for 24 h. The scale bar is 20 μm.

**Fig. S20.** Measurement of hemolysis percentages in the concentration range of 2.5–50 μg/mL DOX and micelles at equivalent DOX concentrations incubated with RBCs at 25°C for 3 h. All data are expressed as the mean ± SD from at least three independent experiments.

|  | \|  \|  \| DOX concentration (μg/mL) \| \| --- \| --- \| --- \| |
| --- | --- | --- | --- | --- |
|  | \| PBS \| Water \| 50 \| 25 \| 10 \| 5 \| 2.5 \| \| --- \| --- \| --- \| --- \| --- \| --- \| --- \| |
| Free DOX + RBC | 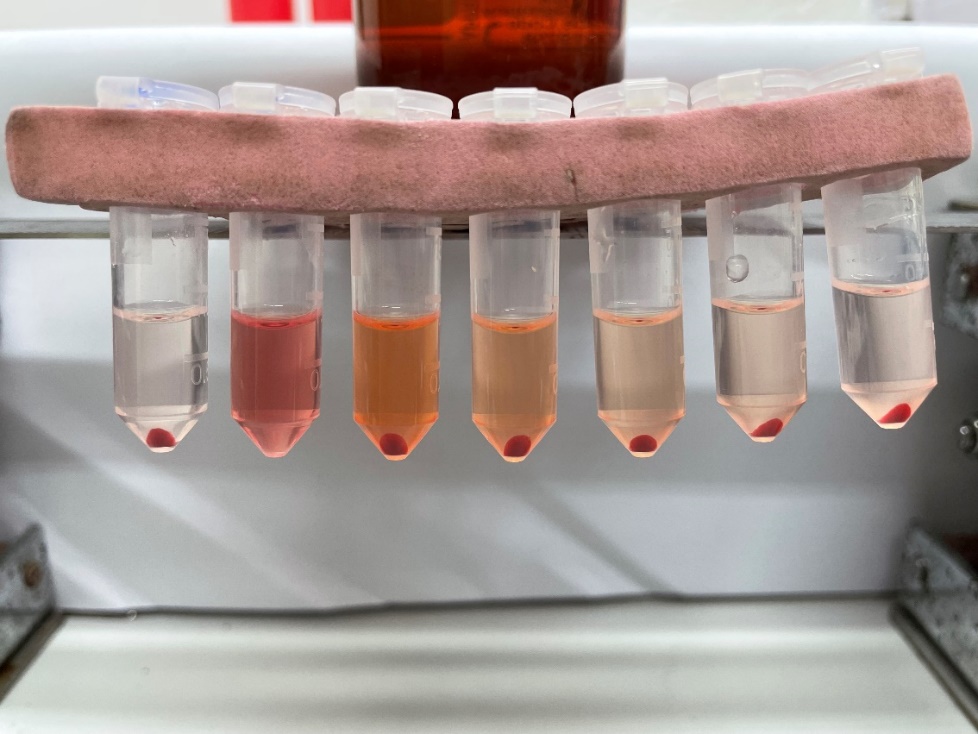 |
| Free DOX | 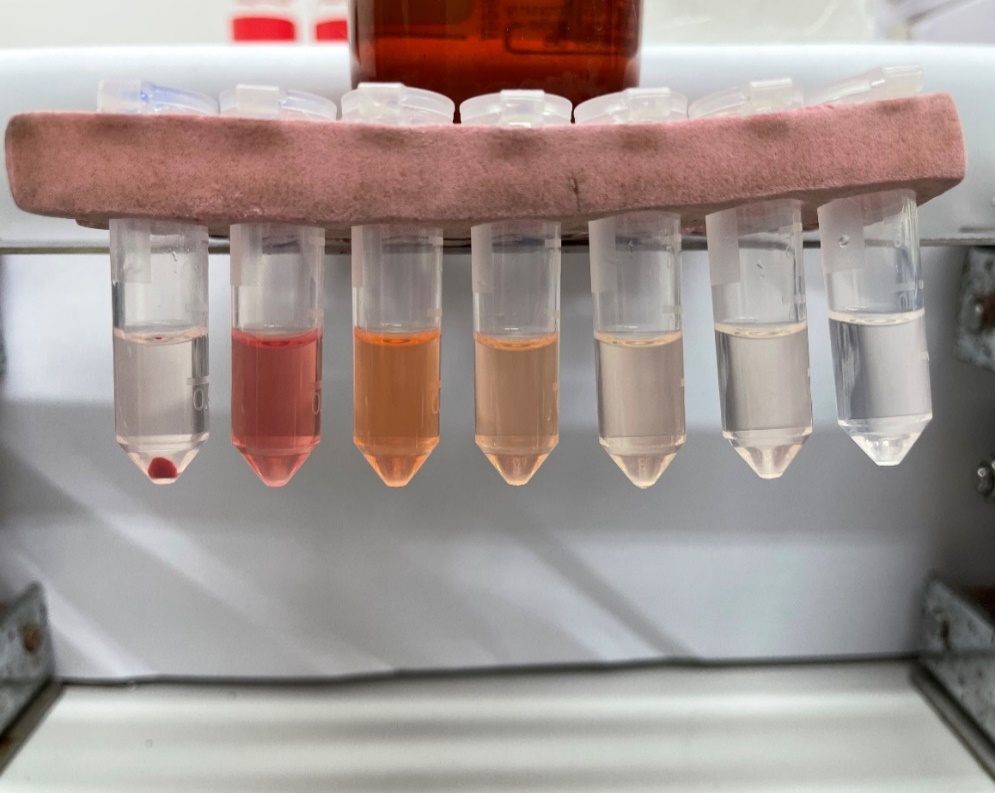 |
| ML-HGD + RBC | 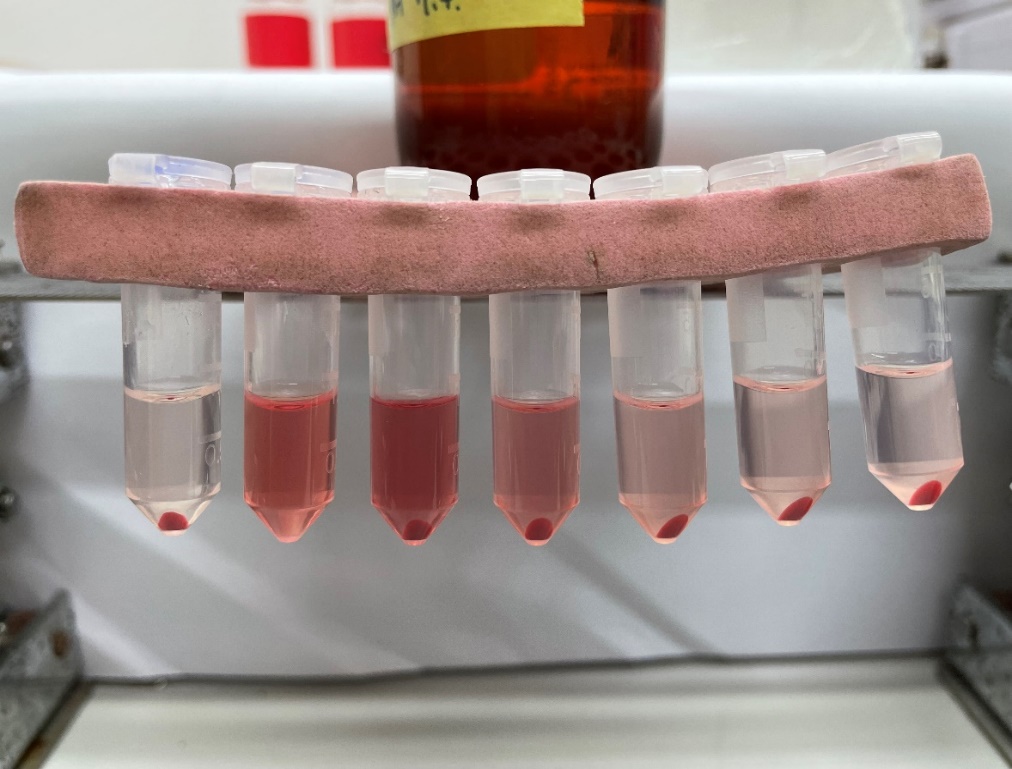 |
| ML-HGD | 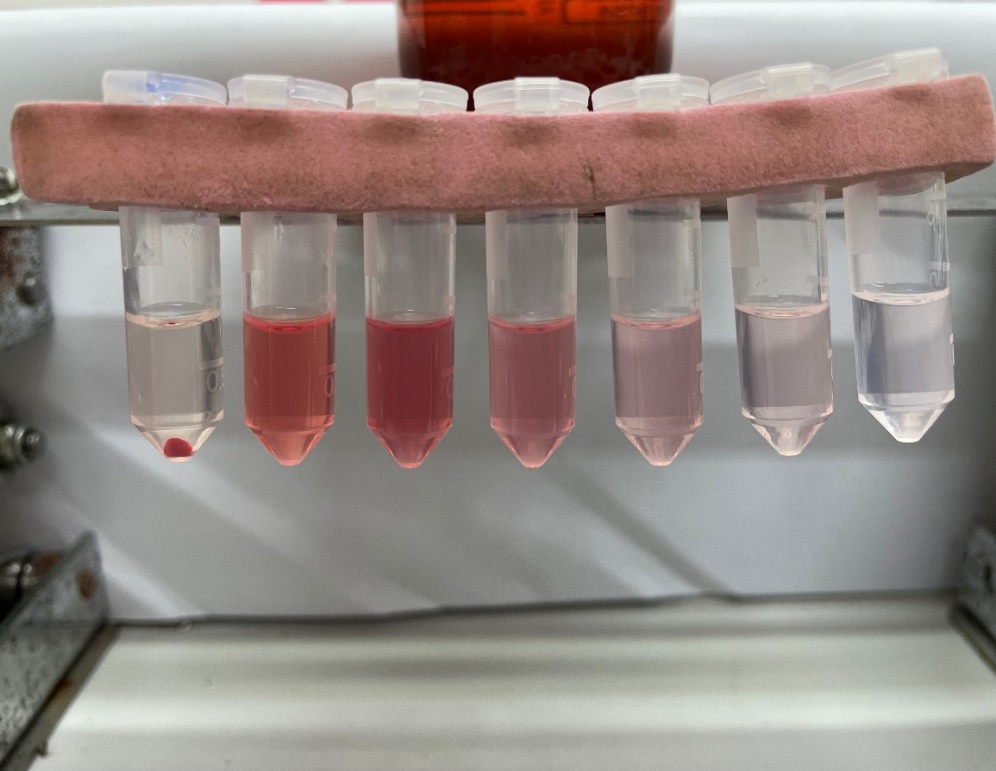 |
| PL-HGD + RBC | 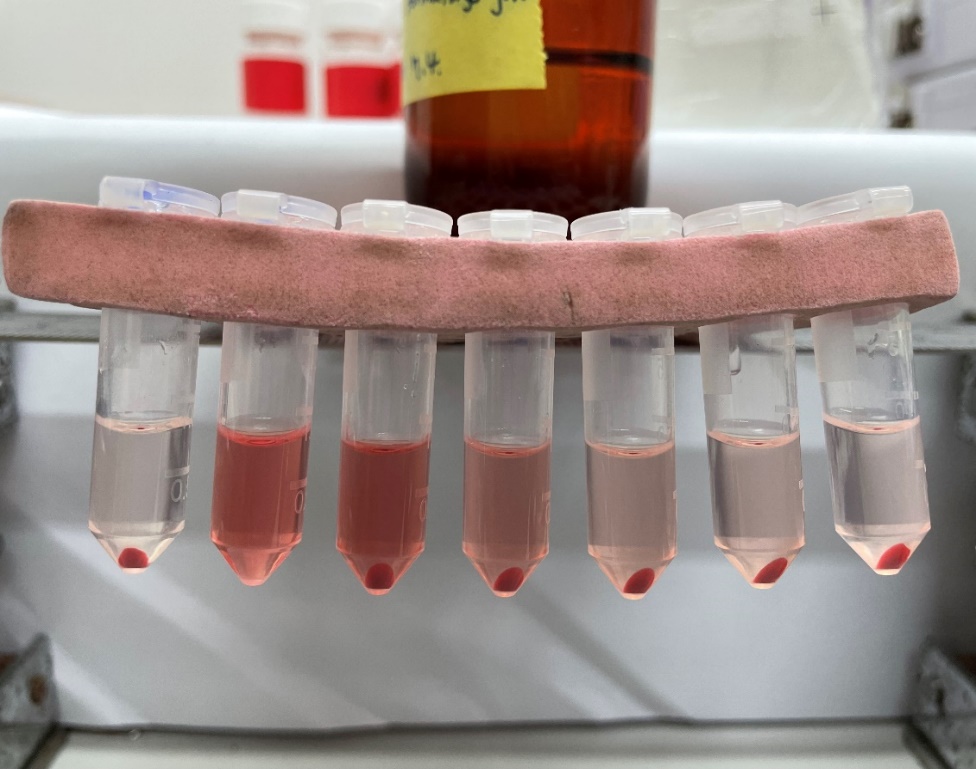 |
| PL-HGD | 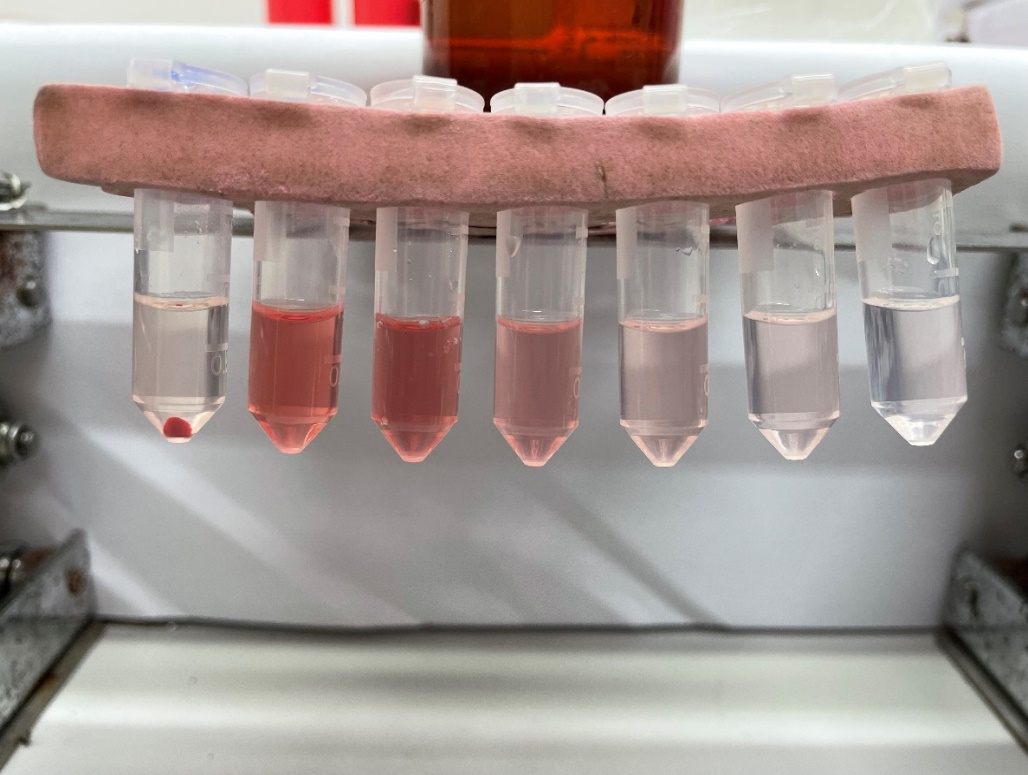 |

**Fig. S21.** Photography of free DOX and micelles at the DOX concentration range from 2.5 to 50 μg/mL incubated with or without mice RBCs. PBS and water served as negative and positive controls, respectively.

| A B |
| --- |
|   |

**Fig. S22.** (A) Glutamic oxaloacetic transaminase (GOT) and (B) creatinine (GRE) measurement for tumor-bearing mice after treatments.

|  | Heart | Lung | Liver | Spleen | Kidney |
| --- | --- | --- | --- | --- | --- |
| Control | 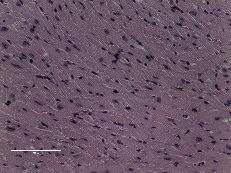 | 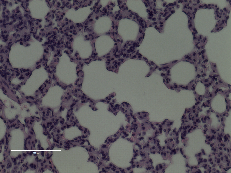 | 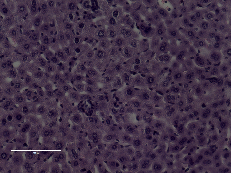 | 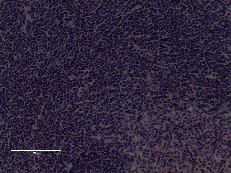 | 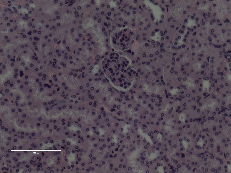 |
| DOX | 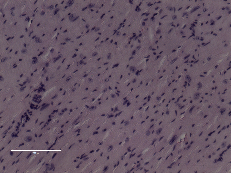 | 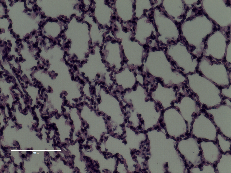 | 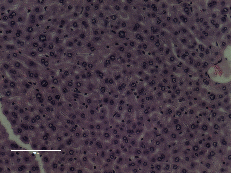 | 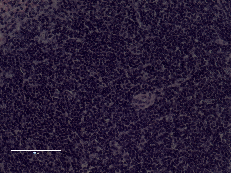 | 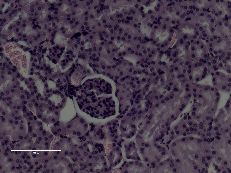 |
| IMQ | 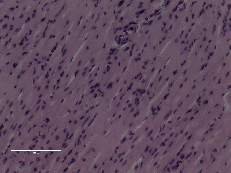 | 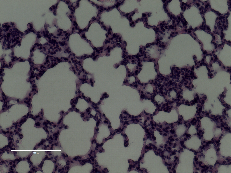 | 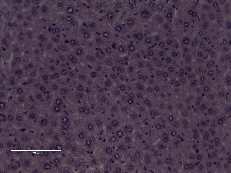 | 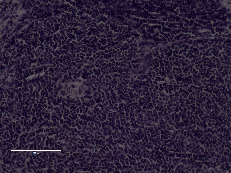 | 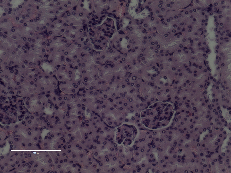 |
| DOX+  IMQ | 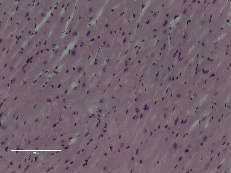 | 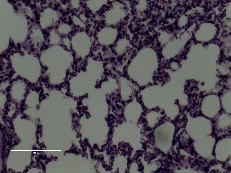 | 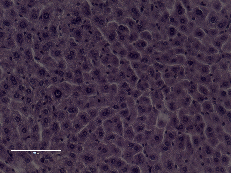 | 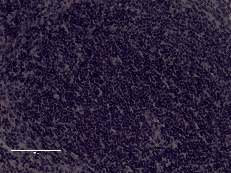 | 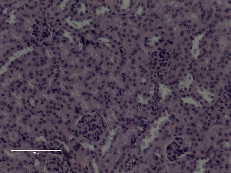 |
| PL-HGD | 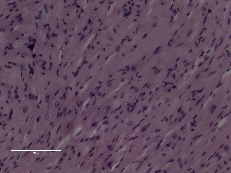 | 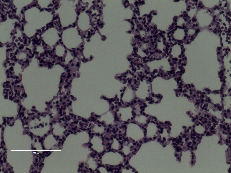 | 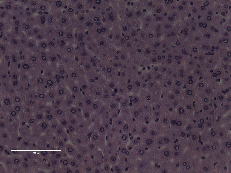 | 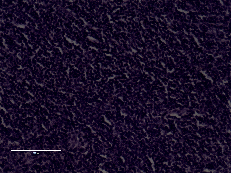 | 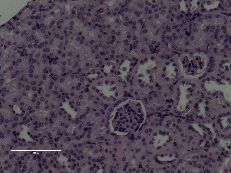 |
| 2XPL-HGD | 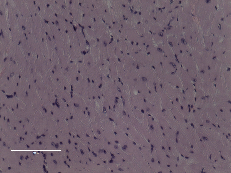 | 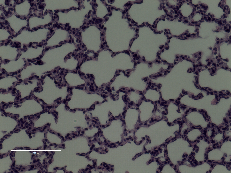 | 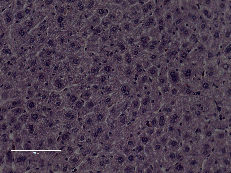 |  |  |

**Fig. S23.** Sections of major organs stained with hematoxylin and eosin (H&E) after treatments. The scale bar indicates 100 μm.

|  | DAPI | iNOS | Merge |
| --- | --- | --- | --- |
| Control |  |  |  |
| DOX + IMQ |  |  |  |
| PL-HGD |  |  |  |
| 2XPL-HGD |  |  |  |

**Fig. S24.** Immunofluorescence image of tumor tissue stained with DAPI and FITC conjugated iNOS. The scale bar is 50 μm
